# *Tenebrio molitor* Spätzle 1b Is Required to Confer Antibacterial Defense Against Gram-Negative Bacteria by Regulation of Antimicrobial Peptides

**DOI:** 10.3389/fphys.2021.758859

**Published:** 2021-11-18

**Authors:** Young Min Bae, Yong Hun Jo, Bharat Bhusan Patnaik, Bo Bae Kim, Ki Beom Park, Tariku Tesfaye Edosa, Maryam Keshavarz, Maryam Ali Mohammadie Kojour, Yong Seok Lee, Yeon Soo Han

**Affiliations:** ^1^Department of Applied Biology, Institute of Environmentally-Friendly Agriculture (IEFA), College of Agriculture and Life Sciences, Chonnam National University, Gwangju, South Korea; ^2^Department of Bio-Science and Bio-Technology, Fakir Mohan University, Balasore, India; ^3^Ethiopian Institute of Agricultural Research, Ambo Agricultural Research Center, Ambo, Ethiopia; ^4^Department of Evolutionary Biology, Institute for Biology–Zoology, Free University of Berlin, Berlin, Germany; ^5^Department of Biology, College of Natural Sciences, Soonchunhyang University, Asan, South Korea

**Keywords:** *T. molitor*, spätzle, innate immunity, antimicrobial peptides, RNA interference

## Abstract

Innate immunity is the ultimate line of defense against invading pathogens in insects. Unlike in the mammalian model, in the insect model, invading pathogens are recognized by extracellular receptors, which activate the Toll signaling pathway through an extracellular serine protease cascade. In the Toll-NF-κB pathway, the extracellular spätzle protein acts as a downstream ligand for Toll receptors in insects. In this study, we identified a novel Spätzle isoform (*Tm*Spz1b) from RNA sequencing database of *Tenebrio molitor*. *Tm*Spz1b was bioinformatically analyzed, and functionally characterized for the antimicrobial function by RNA interference (RNAi). The 702 bp open reading frame of *Tm*Spz1b encoded a putative protein of 233 amino acid residues. A conserved cystine-knot domain with seven cysteine residues in *Tm*Spz1b was involved in three disulfide bridges and the formation of a spätzle dimer. *TmSpz1b* was mostly expressed in the hemocytes of *T. molitor* late instar larvae. The mRNA expression of *TmSpz1b* was highly induced in the hemocytes after *Escherichia coli*, *Staphylococcus aureus*, and *Candida albicans* stimulation of *T. molitor* larvae. *TmSpz1b* silenced larvae were significantly more susceptible to *E. coli* infection. In addition, RNAi-based functional assay characterized *Tm*Spz1b to be involved in the positive regulation of antimicrobial peptide genes in hemocytes and fat bodies. Further, the *TmDorX2* transcripts were downregulated in *TmSpz1b* silenced individuals upon *E. coli* challenge suggesting the relationship to Toll signaling pathway. These results indicate that *Tm*Spz1b is involved in the *T. molitor* innate immunity, causes the sequestration of Gram-negative bacteria by the regulatory action of antimicrobial peptides, and enhances the survival of *T. molitor* larvae.

## Introduction

Innate immune responses, such as antimicrobial peptide (AMP) production, coagulation, prophenoloxidase cascade, phagocytosis, melanization, nodule formation, and encapsulation processes, are the major defense systems against invading pathogens in invertebrates. AMP production is one of the most important innate immune responses. The Toll and immune deficiency (IMD) signaling pathways are the major immune responses that regulate the production of AMPs in *Drosophila* (De Gregorio et al., 2002).

The Toll receptor was first identified in *Drosophila melanogaster* and was reported to be essential for establishment of the dorsal-ventral patterning in the *Drosophila* embryo. The precursor form of the Toll receptor reportedly converts to an active Toll receptor in a position-dependent manner, relative to the dorsal-ventral axis (Anderson et al., 1985). Since 1995, various research groups have studied the effects of the Toll signaling pathway on innate immune responses against various pathogens. In the *Drosophila* model, the dorsal gene, a homolog of a rel-related gene acting as a nuclear factor-kappa B (NF-κB), promotes expression of an antifungal peptide, diptericin, through a Toll signaling pathway, defined by Toll or cactus mutant screening (Lemaitre et al., 1995). In addition, the dorso-ventral regulatory gene cassette (*spätzle*-*Toll*-*cactus*) is involved in the antifungal immune response by regulating the expression of the antifungal peptide gene drosomycin (Lemaitre et al., 1996). Further, it has been suggested that Gram-positive bacteria recognized by the peptidoglycan recognition protein (PGRP) activate the *Drosophila* Toll pathway (Michel et al., 2001). Moreover, an active form of the spätzle cytokine directly binds to the multimerized Toll receptors to initiate the intracellular Toll signaling pathway (Weber et al., 2003; Hu et al., 2004). Interestingly, recent studies have suggested that the Toll signaling pathway is also required for antiviral immune response against oral infection (Ferreira et al., 2014). In addition, *Drosophila* antiviral autophagy against vesicular stomatitis virus (VSV) is triggered by the Toll-7 receptor on the plasma membrane (Nakamoto et al., 2012).

The functional role of Toll-like receptors (TLRs) has been well characterized in mammals. In humans, TLRs, which are the homologs of *Drosophila* Toll receptor, were identified as type I transmembrane proteins that possess an extracellular leucine-rich repeat (LRR) domain that recognizes pathogen associated molecular patterns (PAMPs), and an intracellular Toll-interleukin-1 receptor (TIR) domain that activates downstream signaling (Medzhitov, 2001; Godfroy et al., 2012). TLRs have been classified into two subgroups based on cellular location and PAMP recognition. TLR1, TLR2, TLR4, TLR5, TLR6, and TLR10 reside on the cell membrane and recognize bacterial cell walls. TLR3, TLR7, TLR8, and TLR9 are expressed in intracellular compartments, like endosomes and target bacterial and viral nucleic acids (Kawai and Akira, 2010). The functions of TLR in innate immune signaling have been fully characterized, scrutinized in mammalian models (Yu et al., 2016; Li et al., 2018) and have been summarized (Nie et al., 2018).

The invertebrate Toll pathway includes an extracellular serine protease cascade. In *Drosophila*, the extracellular ligand for Toll pathway, spätzle, is activated during development by two different enzymes, including Easter (Chasan and Anderson, 1989) and the spätzle processing enzyme (SPE), which are required for innate immunity (Jang et al., 2006). Mature spätzle is important in dorsal-ventral polarity (Schneider et al., 1994; Morisato, 2001) and is required for antifungal immune response in *Drosophila* (Lemaitre et al., 1996). A recent study showed that the SPE can be activated by injection of *Micrococcus luteus* and *Bacillus subtilis* (Yamamoto-Hino and Goto, 2016). Furthermore, the spätzle protein secreted from hemocytes regulates the production of AMPs from fat bodies by septic injury (Shia et al., 2009). The spätzle protein is activated by an extracellular serine protease cascade, and the dimeric active form of spätzle (C106) directly binds to the Toll receptor (Weber et al., 2003; Arnot et al., 2010). Various studies have characterized the innate immune functions of spätzle in other insects. In *Aedes aegypti*, spätzle1C activates the Toll5A receptor to mediate an antifungal immune response against the entomopathogenic fungus *Beauveria bassiana* (Shin et al., 2006). In *Bombyx mori*, the active form of recombinant spätzle1 (*Bm*Spz1) protein regulates several AMPs, such as *attacin*, *cecropin*, *gloverin, moricin*, and *lebocin*, unlike the inactive form of recombinant *Bm*Spz1 (Wang et al., 2007). Cleavage of the Spätzle-C108 dimer by extracellular proteolytic cascades activates the Toll pathway in response to a wide variety of microbes. This results in lysozyme stimulation and the production of several AMPs, including *attacin-1*, *cecropin-6*, and *moricin*, in *Manduca sexta* (An et al., 2010). In another lepidopteran insect, *Antheraea pernyi*, the induction patterns of Toll pathway-related genes, including those for Gram-negative bacteria binding protein (GNBP), spätzle1, Toll, MyD88, Cactus, and dorsalA, were analyzed after microbial challenges. The Toll pathway-related genes were significantly induced by the injection of fungi (*Nosema pernyi*) and Gram-positive bacteria (*Enterococcus pernyi*), but not by Gram-negative bacteria (*Escherichia coli*) (Sun et al., 2016). In the mealworm beetle *T. molitor*, *Tm*Spz4 and *Tm*Spz6 are required for the regulation of AMP production against *E. coli*, *C. albicans*, and *S. aureus* infections, suggesting the involvement of AMP in the increased survival of *T. molitor* threatened with infections (Edosa et al., 2020a,b).

In aquatic invertebrates, such as the marine shrimp *Fenneropenaeus chinensis*, the spätzle (*Fc*Spz) gene is induced by the Gram-negative bacterium *Vibrio anguillarum* and white spot syndrome virus (WSSV) (Shi et al., 2009). Furthermore, injection of the active form of *Fc*Spz can induce several AMP genes in crayfish (Shi et al., 2009). In another marine shrimp, *Artemia sinica*, a full-length cDNA sequence of the spätzle gene belonging to spätzle-4 family was identified. The gene was highly induced by injection of Gram-positive bacteria, such as *Micrococcus lysodeikticus*, suggesting an important function in innate immunity (Zheng et al., 2012). In *Macrobrachium rosenbergii*, microbial susceptibility against the Gram-negative bacterium *Aeromonas caviae* was significantly increased by silencing of *Mr*Spz in shrimp (Vaniksampanna et al., 2019). The first mollusk spätzle homolog gene was identified in the clam *Paphia undulate* and was shown to be involved in the host defense against both Gram-negative (*V. alginolyticus*) and Gram-positive bacteria (*Listeria monocytogenes*) (Yu et al., 2015).

In the beetle model, the serine protease signaling cascade for extracellular Toll signaling pathway has been fully characterized by elegant studies using biochemical and molecular approaches. Lysine-type peptidoglycan (PG) recognition complex initially recognizes pathogenic patterns, followed by a three-step proteolytic cascade that finally cleaves the spätzle protein to activate a PG-dependent Toll signaling pathway (Kim et al., 2008; Ali Mohammadie Kojour et al., 2020). Moreover, the fungal cell wall component β-1, 3-glucan also activates the *T. molitor* Toll pathway (Roh et al., 2009). In our recent study on RNA interference (RNAi)-based functional characterization of immune-related genes revealed that *Tm*Cactin, a *Tenebrio* cactus binding protein, was activated by Gram-negative and Gram-positive bacteria, and promiscuously regulated five AMP genes (Jo et al., 2017). Another component of the Toll signaling pathway, *Tm*Toll-7, was interestingly activated by the Gram-negative bacterium, *E. coli*, and positively regulated seven AMP genes (Park et al., 2019). In addition, our recent bioinformatics analysis identified nine spätzle isoforms from the *T. molitor* model (*TmSpz-like*, *-1b*, *-3*, *-4*, *-5*, *-6*, *-7*, *-7a*, *-7b*). Apart from Spätzle4 and Spätzle6 that has been discussed in the context of humoral immunity in *T. molitor* the functions of other spätzle isoforms are still elusive. *Tm*Spz4 regulates AMP production against *E. coli* and *C. albicans* infection through the activation of Toll pathway (Edosa et al., 2020a) and *Tm*Spz6 regulates AMP expression and increases survival of *T. molitor* against *E. coli* and *S. aureus* (Edosa et al., 2020b).

In the present study, a novel spätzle isoform, *TmSpz1b*, was functionally characterized for its function in regulating AMP production against Gram-negative bacteria, but not against Gram-positive bacteria and fungus. The downregulated *TmSpz1b* transcript resulted in high cumulative mortality of *E. coli-*infected *T. molitor* larvae. The results suggest that *Tm*Spz1b is involved in *T. molitor* innate immunity, causing the sequestration of Gram-negative bacteria by the regulatory action of antimicrobial peptides, and enhances survival of *T. molitor* larvae.

## Materials and Methods

### Insect Rearing

Larvae of the yellow mealworm beetle (*T. molitor*) were reared under continuous dark conditions at 27 ± 1°C and 60% ± 5% relative humidity (R.H.) in an environmental chamber. The reared larvae were fed an artificial diet consisting of 170 g wheat flour, 20 g roasted soy flour, 10 g protein, 100 g wheat bran, 0.5 g sorbic acid, 0.5 mL propionic acid, and 0.5 g chloramphenicol in 200 mL of distilled water (D.W.). Only 10th to 12th instar *T. molitor* larvae were used in these experiments.

### Preparation of Microorganisms

The Gram-negative bacterium *E. coli* (strain K12), Gram-positive bacterium *S. aureus* (strain RN4220), and the fungus *C. albicans* (strain AUMC 13529) were used for the immune challenge studies. *E. coli* and *S. aureus* were cultured overnight in Luria-Bertani broth (MB Cell, Seoul, Korea) at 37°C. *C. albicans* was cultured overnight at 37°C in Sabouraud dextrose broth (MB Cell). The microorganisms were harvested and washed twice in 1 × phosphate-buffered saline (PBS; 8.0 g NaCl, 0.2 g KCl, 1.42 g Na_2_HPO_4_, and 0.24 g KH_2_PO_4_ in 1 l of D.W; pH 7.0), and centrifuged at 3,500 rpm for 10 min. Each cell pellet was subsequently suspended in PBS, and the concentrations of microorganisms were measured by their optical density at 600 nm (OD_600_) by spectrophotometry (Eppendorf, Hamburg, Germany). The suspensions were adjusted to 1 × 10^6^ cells/μl (*E. coli* and *S. aureus*) or 5 × 10^4^ cells/μl (*C. albicans*) for immune challenge studies.

### Identification and *in silico* Analysis of *T. molitor Spätzle1b*

To identify the *TmSpz1b* gene (Accession no. MZ708791), a local-tblastn analysis was performed using the amino acid sequence of *Tribolium castaneum* spätzle X3 (*Tc*SpzX3) (GenBank: XP015840683.1) as a query against the locally curated *T. molitor* nucleotide database derived from *T. molitor* RNA sequencing. The deduced amino acid sequence of *Tm*Spz1b was analyzed using the blastx and blastp algorithms (Mount, 2007) at NCBI. The full-length target open reading frame (ORF) region were amplified by AccuPower Pfu Pre-Mix (Bioneer, Daejeon, South Korea) on a MyGenie 96 thermal block (Bioneer) using gene-specific primers designed using Primer 3.0 software^1^ (Table 1). The PCR purified products were cloned into the T-blunt vector cloning system (Solgent Company, Daejeon, South Korea), transformed into *E. coli* DH5α cells, and sequenced using M13 primers. After sequencing the full-length ORF sequence was validated.

**TABLE 1:** Primers used in the study.

Name	Primer sequences
*Tm*Spz1b_cloning_Fw *Tm*Spz1b_cloning_Rv	5′-TACAGGTCAACCCCAAGACC-3′ 5′-CGACGGCACTTTAAACGAAT-3′
*Tm*Spz1b_qPCR_Fw *Tm*Spz1b_qPCR_Rv	5′-GGACGCTTCCCATTAGTGCT-3′ 5′TCTAAGTGTGAATGCGCCGT-3′
*Tm*Spz1b_T7_Fw *Tm*Spz1b_T7_Rv	5′-TAATACGACTCACTATAGGGT GCTGGCTACCCAAAAGAACA-3′ 5′-TAATACGACTCACTATAGGGT CGACGGCACTTTAAACGAAT-3′
*EGFP*_T7_Fw *EGFP*_T7_Rv	5′-TAATACGACTCACTATAGGGT CGTAAACGGCCACAAGTTC -3′ 5′-TAATACGACTCACTATAGGGT TGCTCAGGTAGTGTTGTCG -3′
*Tm*Tenecin-1_qPCR_Fw *Tm*Tenecin-1_qPCR_Rv	5′-CAGCTGAAGAAATCGAACAAGG-3′ 5′-CAGACCCTCTTTCCGTTACAGT-3′
*Tm*Tenecin-2_qPCR_Fw *Tm*Tenecin-2_qPCR_Rv	5′-CAGCAAAACGGAGGATGGTC-3′ 5′-CGTTGAAATCGTGATCTTGTCC-3′
*Tm*Tenecin-3_qPCR_Fw *Tm*Tenecin-3_qPCR_Rv	5′-GATTTGCTTGATTCTGGTGGTC-3′ 5′-CTGATGGCCTCCTAAATGTCC-3′
*Tm*Tenecin-4_qPCR_Fw *Tm*Tenecin-4_qPCR_Rv	5′-GGACATTGAAGATCCAGGAAAG-3′ 5′-CGGTGTTCCTTATGTAGAGCTG-3′
*Tm*Defensin_qPCR_Fw *Tm*Defensin_qPCR_Rv	5′-AAATCGAACAAGGCCAACAC-3′ 5′-GCAAATGCAGACCCTCTTTC-3′
*Tm*Defensin-like_qPCR_Fw *Tm*Defensin-like_qPCR_Rv	5′-GGGATGCCTCATGAAGATGTAG-3′ 5′-CCAATGCAAACACATTCGTC-3′
*Tm*Coleoptericin-A_qPCR_Fw *Tm*Coleoptericin-A_qPCR_Rv	5′-GGACAGAATGGTGGATGGTC-3′ 5′-CTCCAACATTCCAGGTAGGC-3′
*Tm*Coleoptericin-B_qPCR_Fw *Tm*Coleoptericin-B_qPCR_Rv	5′-CAGCTGTTGCCCACAAAGTG-3′ 5′-CTCAACGTTGGTCCTGGTGT-3′
*Tm*Attacin-1a_qPCR_Fw *Tm*Attacin-1a_qPCR_Rv	5′-AAAGTGGTCCCCACCGATTC-3′ 5′-GCGCTGAATGTTTTCGGCTT-3′
*Tm*Attacin-1b_qPCR_Fw *Tm*Attacin-1b_qPCR_Rv	5′-GAGCTGTGAATGCAGGACAA-3′ 5′-CCCTCTGATGAAACCTCCAA-3′
*Tm*Attacin-2_qPCR_Fw *Tm*Attacin-2_qPCR_Rv	5′-AACTGGGATATTCGCACGTC-3′ 5′-CCCTCCGAAATGTCTGTTGT-3′
*Tm*Cecropin-2_qPCR_Fw *Tm*Cecropin-2_qPCR_Rv	5′-TACTAGCAGCGCCAAAACCT-3′ 5′-CTGGAACATTAGGCGGAGAA-3′
*TmTLP1*_qPCR_Fw *TmTLP1*_qPCR_Rv	5′-CTCAAAGGACACGCAGGACT-3′ 5′-ACTTTGAGCTTCTCGGGACA-3′
*TmTLP2*_qPCR_Fw *TmTLP2*_qPCR_Rv	5′-CCGTCTGGCTAGGAGTTCTG-3′ 5′-ACTCCTCCAGCTCCGTTACA-3′
*Tm*DorX1_qPCR_Fw *Tm*DorX1_qPCR_Rv	5′-AGCGTTGAGGTTTCGGTATG-3′ 5′-TCTTTGGTGACGCAAGACAC-3′
*Tm*DorX2_qPCR_Fw *Tm*DorX2_qPCR_Rv	5′-ACACCCCCGAAATCACAAAC-3′ 5′-TTTCAGAGCGCCAGGTTTTG-3′
*Tm*Relish_qPCR_Fw *Tm*Relish_qPCR_Rv	5′-AGCGTCAAGTTGGAGCAGAT-3′ 5′-GTCCGGACCTCAAGTGT-3′
*Tm*L27a_qPCR_Fw *Tm*L27a_qPCR_Rv	5′-TCATCCTGAAGGCAAAGCTCCAGT-3′ 5′-AGGTTGGTTAGGCAGGCACCTTTA-3′

*Underline indicates T7 promotor sequences.*

### Domain and Phylogenetic Analyses

The domain architecture of the protein sequences were retrieved using the InterProScan 5.0 (Jones et al., 2014) and blastp (Mount, 2007) programs. The signal peptide was predicted using the SignalP 5.0 server^2^. The expasy server tools at Swiss Institute of Bioinformatics^3^, including ‘Compute Pi/MW’ and ‘ProtParam,’ were used to identify the physico-chemical properties of the putative protein. The NetPhos 3.1 prediction tool at https://services.healthtech.dtu.dk/ was used to predict serine, threonine, or tyrosine phosphorylation sites in the *Tm*Spz1b protein.

Multiple sequence alignment profile was used to estimate the genetic relatedness of *Tm*Spz1b among Spätzle genes representing different insect orders obtained from GenBank using ClustalX v 2.1 (Larkin et al., 2007) software. Only the amino acid sequence of specific cystine-knot cytokine domain of *Tm*Spz1b was used. The .pim output files from ClustalX v. 2.1 were used to analyze the percentage identity among the insect spätzle sequences from orthologous species. A phylogenetic tree was constructed based on the amino acid sequences of the *TmSpz1b* gene by using the neighbor-joining (NJ) method in the MEGA v. 7.0 software program (Kumar et al., 2016). The bootstrap consensus tree was inferred from 1000 replicates, and the evolutionary distances were computed using the Poisson correction method. The amino acid sequence of *Penaeus vannamei* spätzle (*Pv*Spz; ROT72693.1) was used as an outgroup for this analysis.

### Developmental, Tissue-Specific Expression, and Induction of *TmSpz1b* mRNA in Response to Pathogenic Challenges

To investigate the developmental expression patterns of *TmSpz1b* mRNA, whole body samples (*n* = 20 for each stage) were collected from young larvae (YL; 10th–12th instar larvae), late instar larvae (LL; 19th–20th instar larvae), pre-pupae (PP), 1–7-day old pupae (P1–P7), and 1–5-day old adults (A1–A5). To investigate the tissue-specific expression profiles, integument (IT), fat body (FB), hemocytes (HC), gut (GT), and Malpighian tubules (MT) were dissected from both late instar larvae and 5-day old adults, and ovary (OV) and testes (TE) were collected only from 5-day old adults of *T. molitor*.

To investigate the expression and induction patterns of *TmSpz1b* mRNA, prepared microorganisms including *E. coli*, *S. aureus*, and *C. albicans*, were injected into 11th–12th instar larvae (*n* = 20) of *T. molitor*. PBS injected *T. molitor* group was used as a mock control. The hemocytes, fat body and gut were dissected at 3, 6, 9, 12, and 24 h post-inoculation of microorganisms. The samples were stored at −80°C for further use.

Total RNA was extracted by a Clear-S^TM^ Total RNA Extraction Kit (Invirustech Co., Gwangju, South Korea). To synthesize cDNA, total RNA (2 μg) was used as the template with an Oligo(dT)_12__–__18_ primer at 72°C for 5 min, 42°C for 1 h, and 94°C for 5 min on a MyGenie96 Thermal Block (Bioneer) and using AccuPower^®^ RT PreMix (Bioneer) according to manufacturer’s instructions. cDNA was stored at −20°C until further use.

The relative expression level of *TmSpz1b* mRNA was investigated by performing quantitative real-time polymerase chain reaction (qRT-PCR) using AccuPower^®^ 2X Greenstar^TM^ qPCR Master Mix (Bioneer), with synthesized cDNAs, and *TmSpz1b* gene-specific primers designed using the Primer 3 plus program^4^, as listed in Table 1. The qRT-PCR was programmed at an initial denaturation of 95°C for 5 min, followed by 40 cycles of denaturation at 95°C for 15 s, and annealing and extension at 60°C for 30 s. The qRT-PCR assays were performed on an AriaMx Real-Time PCR System (Agilent Technologies, Santa Clara, CA, United States), and the results were analyzed using AriaMx Real-Time PCR software. *T. molitor ribosomal protein L27a* (*TmL27a*) was used as an internal control, and the mRNA expression levels were analyzed by using 2^–ΔΔCt^ methods (Livak and Schmittgen, 2001). The results represent mean ± SE of three biological replications.

### Synthesis of Double-Stranded RNA

Double-stranded RNAs (dsRNA) for the *TmSpz1b* gene were synthesized to perform RNAi experiments. For the synthesis of dsRNA, the *TmSpz1b* DNA fragment was amplified by PCR using gene-specific primers tailed (5′ end) with a T7 promoter sequence (Table 1). The primers were designed using SnapDragon software^5^ to prevent any cross-silencing effects. PCR products were amplified using AccuPower^®^ Pfu PCR PreMix under the following cycling conditions: an initial denaturation step at 94°C for 5 min, followed by 30 cycles of denaturation at 94°C for 30 s, annealing at 53°C for 40 s, and extension at 72°C for 40 s on a MyGenie96 Thermal Block (Bioneer). The PCR products were purified by the AccuPrep PCR Purification Kit (Bioneer), and dsRNA was synthesized from purified PCR products (1 μg) using the EZ^TM^ T7 High Yield *in Vitro* Transcription Kit (Enzynomics, Daejeon, South Korea), according to the manufacturer’s instructions. The dsRNA product was purified by the Phenol: Chloroform: Isoamyl alcohol mixture (PCI) method, precipitated with 5 M ammonium acetate, and washed with 70 and 90% ethanol. Subsequently, it was quantified using an Epoch spectrophotometer (BioTek Instruments, Inc., Winooski, VT, United States). The synthesized dsRNA was stored at −20°C until further use.

For the knockdown validation of *TmSpz1b* mRNA, 1 μg/μl of synthesized dsRNA of *Enhanced green fluorescent protein* (*EGFP*) and *TmSpz1b* were injected into *T. molitor* young-instar larvae (10th–12th instars; *n* = 20) by using disposable capillary needles mounted on a micro-applicator (Picospiritzer III Micro Dispense System; Parker Hannifin, Hollis, NH, United States). EGFP dsRNA synthesized from pEGFP-C1 plasmid DNA was used as a negative control for RNAi.

### Mortality Assay

To measure the cumulative mortality in *TmSpz1b* knockdown *T. molitor* larvae, healthy larvae were injected with 1 μg/μl of ds*TmSpz1b* or ds*EGFP*. Subsequently, *E. coli*, *S. aureus* and *C. albicans* were injected into *TmSpz1b* silenced *T. molitor* larvae. Dead larvae were counted daily for up to 10 days. Ten insect larvae were used for each set of mortality assays, and the experiments were repeated in triplicate. The results were obtained by Kaplan–Meier survival analysis (Goel et al., 2010).

### Effect of *TmSpz1b* RNAi on Antimicrobial Peptide Gene Expression

To further characterize the immunological function of *TmSpz1b* gene in humoral innate immune response, the effect of *TmSpz1b* silencing by RNAi on the expression levels of 14 AMP genes against microbial challenge were investigated. Two days post-treatment of *TmSpz1b* dsRNA into *T. molitor* larvae, these larvae were injected with *E. coli* or *S. aureus* (1 × 10^6^ cells/larva), or *C. albicans* (5 × 10^4^ cells/larva). After 24 h, immune organs that included hemocytes, fat bodies, and the gut were dissected. Total RNA was extracted, and cDNA was synthesized as described above. The ds*EGFP*-treated *T. molitor* larvae and PBS were used as the negative and injection controls, respectively.

Expression patterns of 14 AMP genes including *TmTenecin1, 2, 3*, and *4* (*TmTene1*, −*2*, −*3*, and −*4*) (Kim et al., 1998; Chae et al., 2012; Yang et al., 2017), *TmDefensin* and *TmDefensin-like* (*TmDef* and *TmDef-like*) (Jang et al., 2020b), *TmColeoptericin-A* and *-B* (*TmColeA* and −*B*) (Zhu et al., 2014; Jang et al., 2020a), *TmAttacin-1a, −1b* and *-2* (*TmAtt1a*, −*1b* and −*2*) (Jo et al., 2018), *TmCecropin-2* (*TmCec2*) (Ali Mohammadie Kojour et al., 2021), and *TmThaumatin*-*like protein-1* and −*2* (*TmTLP1* and −*2*) (Noh and Jo, 2016; Kim et al., 2017), were examined by qRT-PCR with the AMP gene-specific primers (Table 1). A relative quantitative PCR was performed as detailed above in an AMP-specific primer.

### Statistical Analysis

The statistical analysis was performed by one-way analysis of variance (ANOVA) and Tukey’s multiple range tests were used to estimate the difference between groups (*p* < 0.05).

## Results

### Identification and *in silico* Analysis of *TmSpz1b* Genes

**FIGURE 1:**
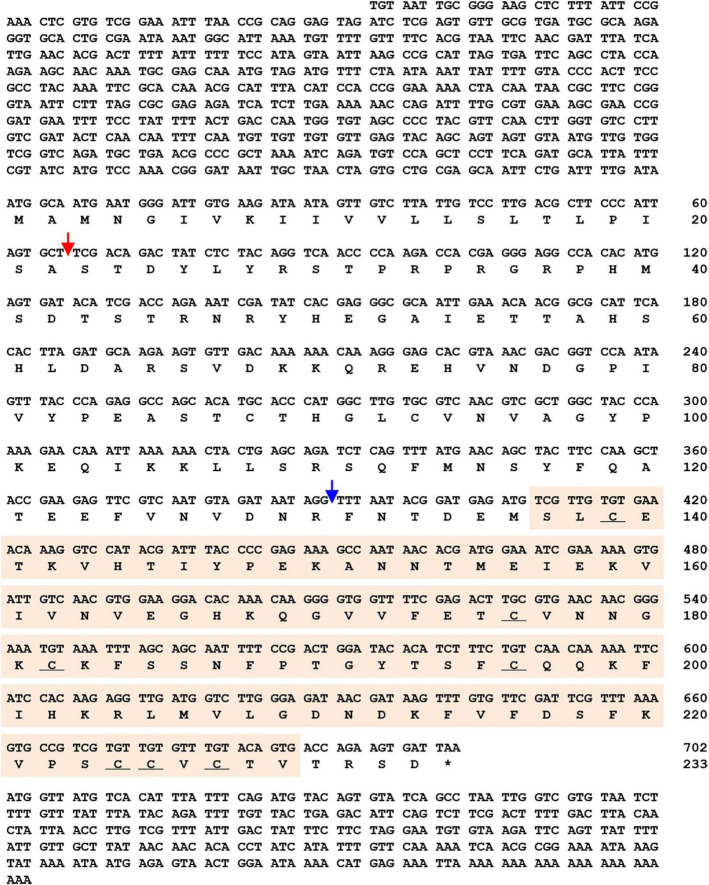
Nucleotide and deduced amino acid sequences of *TmSpz1b*. *TmSpz1b* includes an ORF sequence of 702 bp encoding a polypeptide of 233 amino acid sequences. The 5′- and 3′-UTRs are 627 bp- and 276 bp long, respectively, excluding the poly-A tail. A polyadenylation signal sequence (5′-AATAAA-3′) is marked with blue text. Nucleotides and amino acids are numbered on the right of the sequences. * Denotes stop codon. Domain analysis of *Tm*Spz1b revealed a C-terminal cystine-knot domain (orange box), a signal peptide region (cleaving site between amino acids 22 and 23, red arrow), and a putative cleavage site (blue arrow). Seven conserved cystine residues (underlined) are located in the cystine-knot domain.

The *TmSpz1b* gene was identified using *in silico* protocols. A tblastn analysis with the amino acid sequence of *T. castaneum* spätzle 1b (XP_975083.1) as query against the *T. molitor* RNA sequencing database was useful to screen *TmSpz1b*. The obtained *TmSpz1b* nucleotide sequence was confirmed by blastx analysis^6^ against the GenBank nr database. The ORF of *TmSpz1b* was confirmed by cloning and sequencing. The full-length cDNA of *TmSpz1b* was 1,650 bp in length, including a 627 bp and 276 bp 5′- and 3′- untranslated region (UTR), respectively, excluding the poly-A tail. A polyadenylation signal (5′-AATAAA-3′) was located 11 bp upstream of the poly-A tail sequence. The 702 bp ORF of *TmSpz1b* encoded a putative protein of 233 amino acids (Figure 1) with a calculated molecular weight of 26.38 kDa and a pI of 8.33. The total number of positively charged residues (Asp + Glu) in *Tm*Spz1b was 25. The total number of negatively charged residues (Arg + Lys) was 28. *Tm*Spz1b had an extinction coefficient of 12,420 (Cys form Cystines) and 11,920 (Cys are reduced), with an instability index, aliphatic index, and grand average of hydropathicity of 35.29 (indicating a stable protein), 69.74, and −0.412, respectively.

To determine the structure of this gene, domain analysis was performed using the blastp and InterProScan 5 programs. The findings indicated that *Tm*Spz1b possesses a C-terminal cystine-knot domain (spätzle superfamily domain; pfam 16077), a signal peptide region (cleavage site between amino acid positions 22 and 23), and a putative cleavage site. In the cystine-knot domain, seven conserved cystine residues were located, forming three disulfide bridges, and one cysteine was involved in the dimerization process. A total of 46 phosphorylation sites were predicted on Ser, Thr, or Tyr residues in *Tm*Spz1b. No glycosylation sites were found.

**FIGURE 2:**
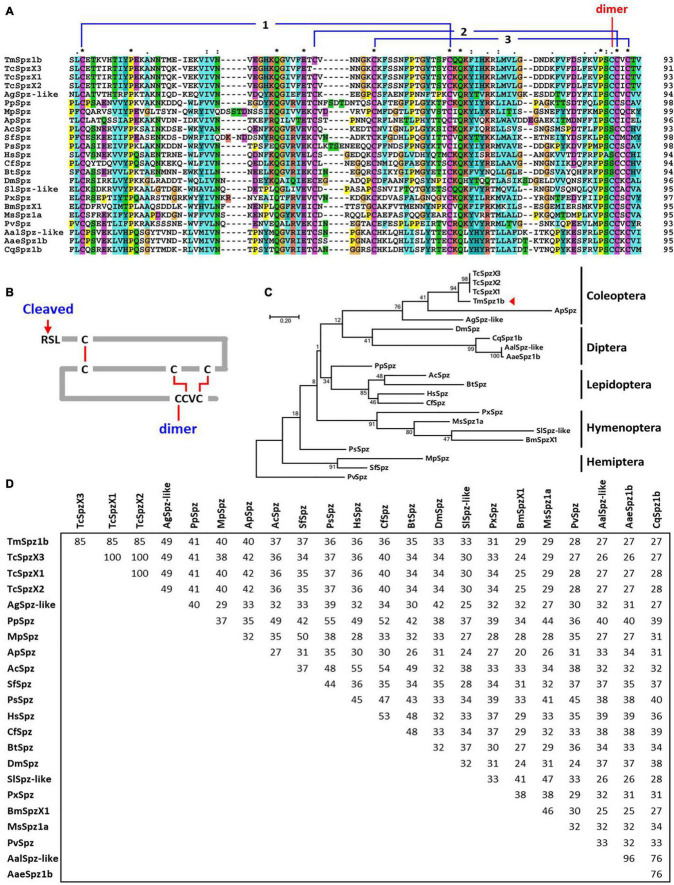
Multiple sequence alignment and phylogenetic analyses of Spätzle proteins. **(A)** Multiple sequence alignment of the spätzle domain in Spätzle proteins. A high degree of conservation is evident in the spätzle domain. The conserved cysteine residues forming the three disulfide bonds are shown. The cysteine involved in the dimer formation is also denoted. **(B)** Deduced cystine-knot domain of *Tm*Spz1b. The six cystine residues form three disulfide bridges, and the one extra cystine residue may interact with the other active form of Spätzle protein. **(C)** Phylogenetic tree of Spätzle proteins. *Tm*Spz1b is located on the same branch occupied by *Tc*SpzX1, *Tc*SpzX2, and *Tc*SpzX3. **(D)** Percentage identity of *Tm*Spz1b with its orthologs. The *Penaeus vannamei* spätzle protein (*Pv*Spz) sequence was used as the outgroup. *Tm*Spz1b, *Tenebrio molitor Spätzle-1b*; *Ag*Spzlike, *Anoplophora glabripennis* protein spaetzle-like (XP_018564206.1); *Ap*Spz, *Agrilus planipennis* protein spaetzle (XP_018334006.1); *Tc*SpzX3, *Tribolium castaneum* PREDICTED: protein spaetzle isoform X3 (XP_015840683.1); *Tc*SpzX1, *Tribolium castaneum* PREDICTED: protein spaetzle isoform X1 (XP_008201187.1); *Bt*Spz, *Bombus terrestris* protein spaetzle (XP_003402363.1); *Hs*Spz, *Harpegnathos saltator* protein spaetzle (XP_011149648.1); *Cf*Spz, *Camponotus floridanus* protein spaetzle (XP_011256297.1); *Ac*Spz, *Apis cerana cerana* Protein spaetzle (PBC29562.1); *Ps*Spz, *Plautia stali* protein spaetzle (BBE08127.1); *Mp*Spz, *Myzus persicae* protein spaetzle-like (XP_022173331.1); *Pp*Spz, *Pristhesancus plagipennis* secreted Spaetzle-like protein (ATU82783.1); *Sf*Spz, *Sipha flava* protein spaetzle (XP_025420977.1); *Cq*Spz1b, *Culex quinquefasciatus* spätzle 1B (XP_001864596.1); *Aal*Spz-like, *Aedes albopictus* protein spaetzle-like (XP_029718352.1); *Aae*Spz1b, *Aedes aegypti* spaetzle1B precursor (NP_001350875.1); *Dm*Spz, *Drosophila melanogaster* spätzle (ABM21577.1); *Px*Spz, *Papilio xuthus* Protein spaetzle (KPJ02943.1); *Sl*Spz-like, *Spodoptera litura* protein spaetzle-like (XP_022825571.1); *Bm*SpzX1, *Bombyx mori* spätzle-1 isoform X1 (XP_021206899.1); *Ms*Spz1a, *Manduca sexta* Spz1A (ACU68553.1); *Pv*Spz, *Penaeus vannamei* protein spaetzle (ROT72693.1).

To understand the evolutionary relationship between *Tm*Spz1b and other insect spätzle proteins, multiple alignment and phylogenetic analyses of insect cystine-knot domains (highly conserved domains of spätzle proteins) were performed using the clustalX2 and MEGA X programs. The multiple alignments of cystine-knot domains showed that seven cystine residues mainly involved in structure formation were well conserved in insects, except Hymenopteran insects (Figure 2A). Domain analysis of *Tm*Spz1b also determined a conserved cystine-knot domain harboring seven cystine residues and a putative cleavage site. In addition, six cystine resides formed three disulfide bridges, with the remaining cystine residue perhaps involved in the formation of an active spätzle dimer (Figure 2B). Phylogenetic analysis indicated that *Tm*Spz1b was located on the same branch with coleopteran insects, including the *T. castaneum* protein spätzle isoforms X1, X2, and X3 (Figure 2C). Approximately 85% sequence identity was shared between *Tm*Spz1b and *Tc*SpzX1, X2, and X3 (Figure 2D).

### Developmental and Tissue-Specific Expression Patterns of *TmSpz1b*

**FIGURE 3:**
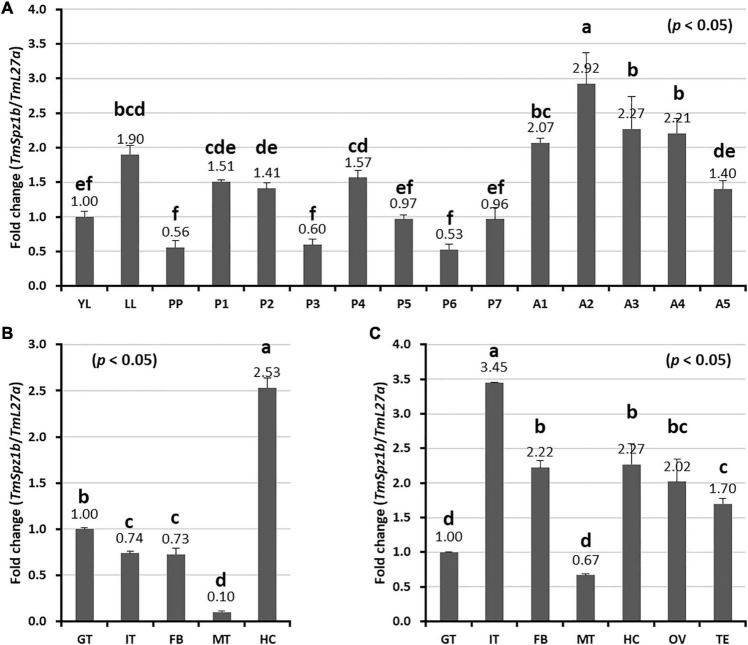
Expression profiles of *TmSpz1b* mRNA during development and in tissues of *T. molitor* assessed by real-time PCR. **(A)** Developmental expression patterns of *TmSpz1b* mRNA. YL, young larvae (10th–12th instar); LL, late instar larvae; PP, pre-pupae; P1–P7, 1- to 7-day-old pupae; and A1–A5, 1- to 5-day-old adults. Tissue-specific expression profiles of *TmSpz1b* were examined in late instar larvae **(B)** and adults **(C)**. IT, integument; GT, gut; FB, fat body; HC, hemocytes; MT, Malpighian tubules; OV, ovary; and TS, testis. Total RNAs extracted from developmental stages and different tissues were reverse transcribed to cDNAs to serve as templates. Vertical bars represent mean ± standard error of three biological replicates. One-way ANOVA and Tukey’s multiple range tests at 95% confidence level (*p* < 0.05) were performed and used to determine the level of significant differences. The graphs indicated by the same letter are not significantly different in Tukey’s multiple range test (*p* < 0.05).

To understand the basic expression patterns of *TmSpz1b*, developmental and tissue-specific expression profiles were investigated by qRT-PCR analysis. *TmSpz1b* was highly expressed at the 2-day-old adult stage. The lowest expression was observed in the prepupal and 3- and 6-day-old pupal stages (Figure 3A). In general, the expression of *TmSpz1b* mRNA was greater in adults and in the late-larval stage. In addition, tissue-specific expression patterns of *TmSpz1b* were examined in late instar larvae (Figure 3B) and 5-day-old adults (Figure 3C). The results indicated that *TmSpz1b* is highly expressed in the hemocytes (2.5-fold) of late instar larvae and integument (approximately 3.5-fold) of 5-day-old adults.

### Induction Patterns of *TmSpz1b*

**FIGURE 4:**
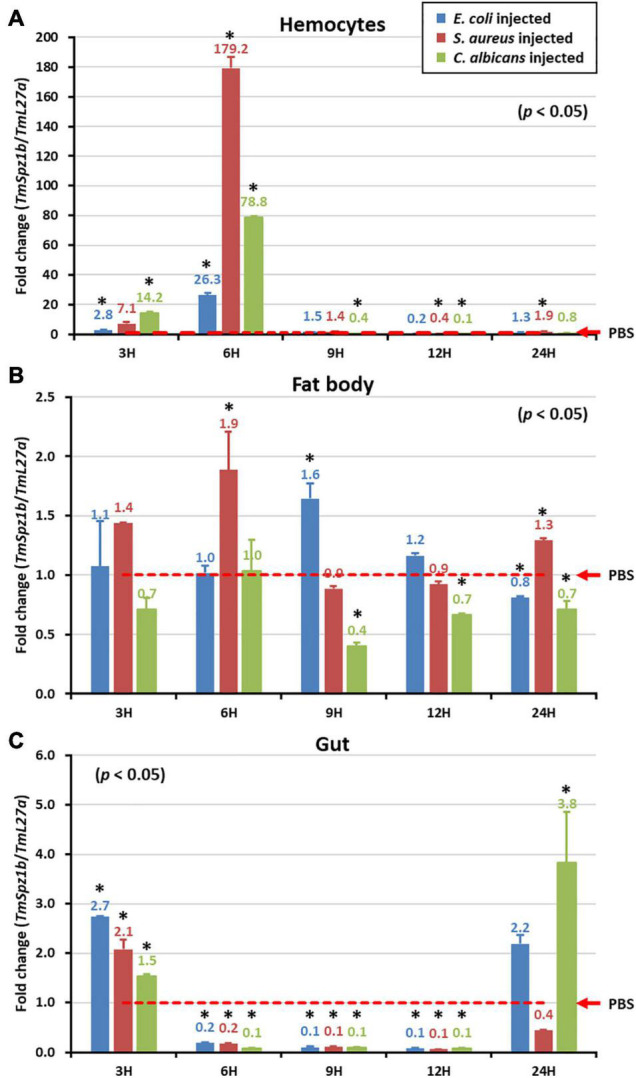
Temporal expression patterns of *TmSpz1b* in three immune organs following microbial challenge. After injection of *E. coli*, *S. aureus*, or *C. albicans*, three immune organs including hemocytes **(A)**, fat bodies **(B)**, and the gut **(C)** were dissected and collected at 3, 6, 9, 12, and 24 h. *TmSpz1b* expression was analyzed by qRT-PCR. PBS-treated group was used as the mock control. The expression level of *TmSpz-like* mRNA in the mock control group was normalized to 1. Vertical bars depict the mean ± S.E. of three biological replicates. Significant differences (*P* < 0.05) between the experimental and control group are indicated by asterisks (^∗^).

To elucidate the putative role of *TmSpz1b* in *Tenebrio* innate immunity, temporal expression patterns of *TmSpz1b* against microbial challenges were investigated in different immune organs. *E. coli* (1 × 10^6^ cells/μl), *S. aureus* (1 × 10^6^ cells/μl), and *C. albicans* (5 × 10^4^ cells/μl) were injected into *T. molitor* 10th to 12th instar larvae. Three immune organs (hemocytes, fat bodies, and the gut), were collected at different times (3, 6, 9, 12, and 24 h). In hemocytes, *TmSpz1b* was dramatically induced at 6 h after injection of *E. coli* (approximately 30-fold), *S. aureus* (approximately 180-fold), and *C. albicans* (80-fold) (Figure 4A). *TmSpz1b* mRNA expression was drastically reduced at later time points after a dramatic increase at 6 h post-infection. However, *TmSpz1b* expression was not strongly induced in the fat bodies and the gut. Interestingly, in the gut, *TmSpz1b* expression was significantly decreased at 6, 9, and 12 h following the injection of microorganisms, compared to that in the PBS injected control group (Figures 4B,C).

### Knockdown of *TmSpz1b* Decreased Larval Survivable Following Microbial Challenges

**FIGURE 5:**
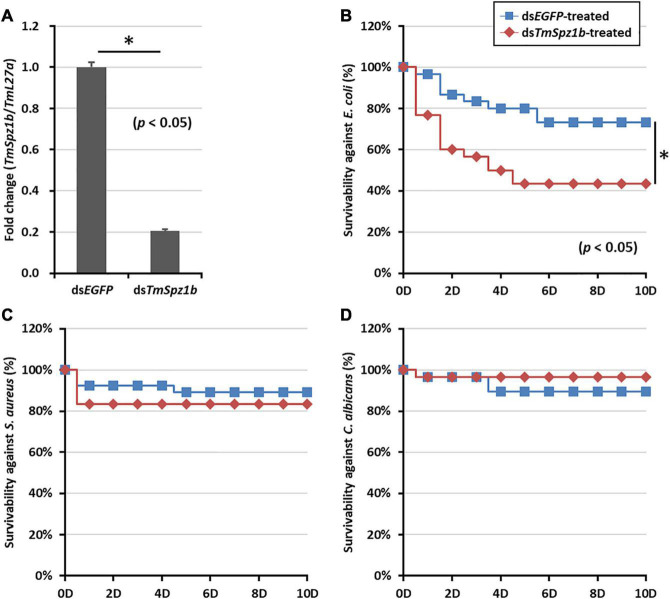
Effects of *TmSpz1b* gene-silencing on larval survival upon microbial challenges. **(A)** Validation of RNAi in ds*TmSpz1b* treated larvae compared with that in dsEGFP-treated larvae. *TmSpz1b* expression was decreased by approximately 80% following the injection of *TmSpz1b*-specific dsRNAs, compared to that in the ds*EGFP-*treated group. *E. coli* **(B)**, *S. aureus* **(C)**, and *C. albicans* **(D)** were injected into *TmSpz1b*-silenced *T. molitor* larvae, and larval survival was monitored for 10 days. The ds*EGFP-*treated groups were used as controls. Survival of larvae infected with *E. coli*, but not *S. aureus* and *C. albicans*, was significantly decreased by *TmSpz1b* knockdown. The data are an average of three biologically independent replicate experiments. Asterisks indicate significant differences between ds*TmSpz4-* and ds*EGFP-*injected groups (*P* < 0.05). Statistical analysis of survival analysis was carried out based on Kaplan–Meier plots (log-rank chi-square test; ^∗^*P* < 0.05).

To assess the function of highly expressed *TmSpz1b* in hemocytes, the effects of *TmSpz1b* RNAi on larval survivable following microbial challenges were investigated. Initially, *TmSpz1b* dsRNA (1 μg/larva) was injected into *T. molitor* larvae. The knockdown ratio was investigated by qRT-PCR analysis. A decrease of *TmSpz1b* expression of approximately 80% (0.2-fold) was observed following injection of *TmSpz1b*-specific dsRNA, compared to that in the ds*EGFP-*treated group (1.0-fold) at 2 days post-injection (Figure 5A).

Following injection of *E. coli*, *S. aureus*, and *C. albicans* into *TmSpz1b*-silenced *T. molitor* larvae, the survival of the larvae was monitored for 10 days. Interestingly, larval survivability upon *E. coli* challenge, but not upon challenge with *S. aureus* and *C. albicans*, was significantly decreased by knockdown of *TmSpz1b*, compared to that in the ds*EGFP*-treated group (Figures 5B–D). In *E. coli* treated *TmSpz1b*-silenced larvae, survival was reduced to 40% and was significantly different from the ds*EGFP*-treated larvae up to 10 days after infection. The survival of *S. aureus-* and *C. albicans*-infected *TmSpz1b*-silenced larvae was reduced by nearly 80% but was not significantly different from the ds*EGFP*-treated groups.

### Effects of *TmSpz1b* RNAi on Expression of 14 Antimicrobial Peptide Genes

**FIGURE 6:**
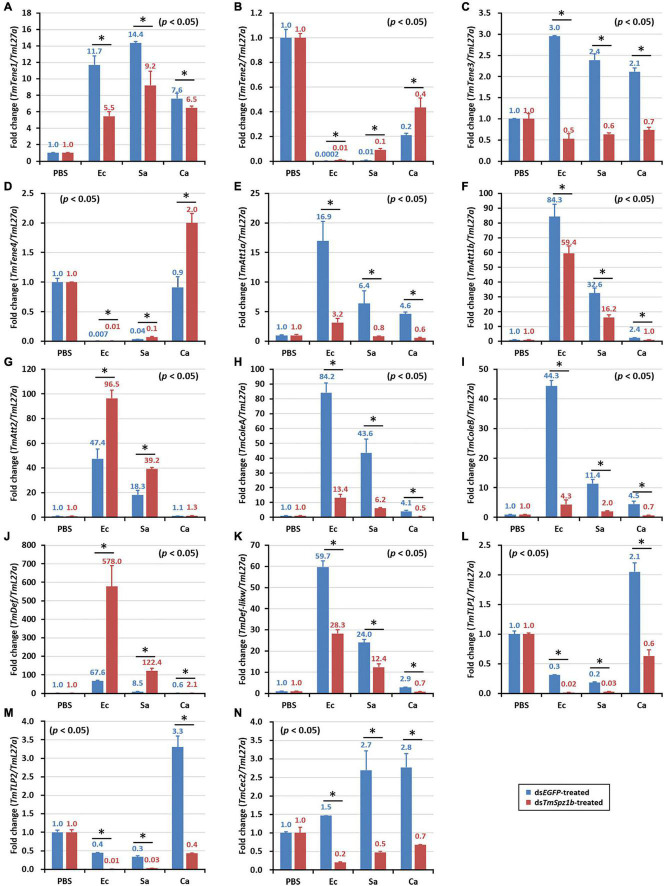
Effects of *TmSpz1b* gene-silencing on the expression of 14 AMP genes in response to pathogen injection in hemocytes. *E. coli, S. aureus*, and *C. albicans* were injected into ds*TmSpz1b*-treated *T. molitor* larvae. The expression levels of 14 AMP genes were determined at 24 h post injection, by qRT-PCR. In hemocytes, seven AMP genes (*TmTenecin1* and *3*, *TmAttacin1a*, and *1b*, *TmColeoptericin1* and *2*, and *TmDefensin2*) were significantly decreased by *TmSpz1b* RNAi. *dsEGFP* was injected as a negative control, and *TmL27a* was used as an internal control. All experiments were performed in triplicate. Asterisks indicate significant differences between ds*TmSpz1b* and ds*EGFP*-treated groups when compared by Student’s *t*-test (*P* < 0.05). *TmTene1* (**A**; *TmTeneecin-1*), *TmTene2* (**B**; *TmTenecin-2*). *TmTene3* (**C**; *TmTenecin-3*), *TmTene4* (**D**; *TmTenecin-4*), *TmAtt1a* (**E**; *TmAttacin-1a*), *TmAtt1b* (**F**; *TmAttacin-1b*), *TmAtt2* (**G**; *TmAttacin-2*), *TmCole1* (**H**; *TmColeoptericin-1*), *TmCole2* (**I**; *TmColeoptericin-2*), *TmDef1* (**J**; *TmDefensin-1*), *TmDef2* (**K**; *TmDefensin-2*), *TmTLP1* (**L**; *TmThaumatin-like protein-1*), *TmTLP2* (**M**; *TmThaumatin-like protein-2*), *TmCec2* (**N**; *TmCecropin-2*).

**FIGURE 7:**
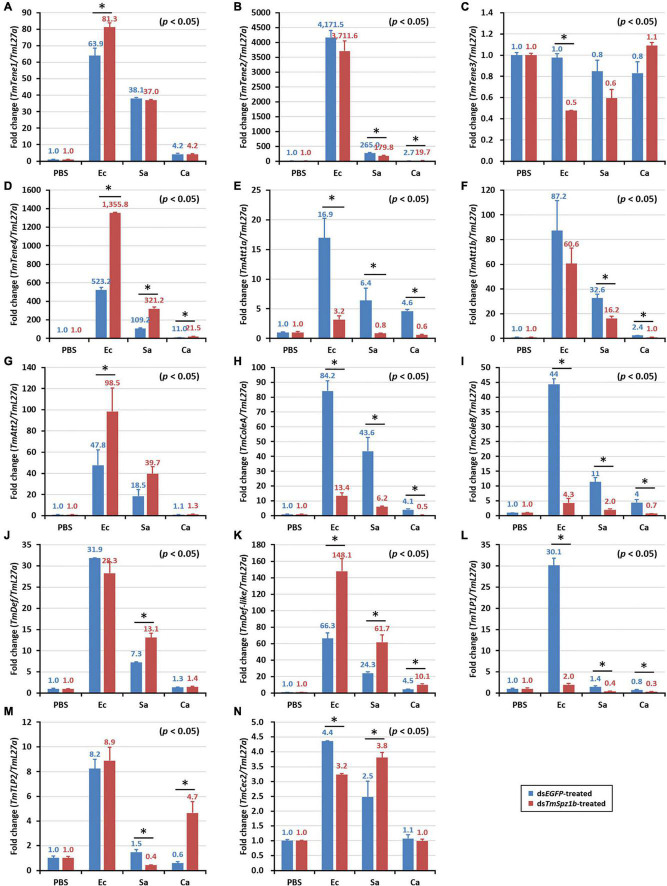
Effects of *TmSpz1b* gene-silencing on the expression of 14 AMP genes in response to pathogen injection in fat body. *E. coli, S. aureus*, and *C. albicans* were injected into ds*TmSpz1b*-treated *T. molitor* larvae. The expression levels of 14 AMP genes were determined at 24 h post injection, by qRT-PCR. In fat bodies, five AMP genes (*TmAttacin1a*, and *1b*, *TmColeoptericin1* and *2*, and *TmTLP1*) were significantly decreased by *TmSpz1b* RNAi. *dsEGFP* was injected as a negative control and *TmL27a* was used as an internal control. All experiments were performed in triplicate. Asterisks indicate significant differences between ds*TmSpz1b* and ds*EGFP*-treated groups when compared by Student’s *t*-test (*P* < 0.05). *TmTene1* (**A**; *TmTeneecin-1*), *TmTene2* (**B**; *TmTenecin-2*). *TmTene3* (**C**; *TmTenecin-3*), *TmTene4* (**D**; *TmTenecin-4*), *TmAtt1a* (**E**; *TmAttacin-1a*), *TmAtt1b* (**F**; *TmAttacin-1b*), *TmAtt2* (**G**; *TmAttacin-2*), *TmCole1* (**H**; *TmColeoptericin-1*), *TmCole2* (**I**; *TmColeoptericin-2*), *TmDef1* (**J**; *TmDefensin-1*), *TmDef2* (**K**; *TmDefensin-2*), *TmTLP1* (**L**; *TmThaumatin-like protein-1*), *TmTLP2* (**M**; *TmThaumatin-like protein-2*), *TmCec2* (**N**; *TmCecropin-2*).

Next, to determine the mechanism of action of *TmSpz1b* gene in the humoral immunity of *T. molitor*, the expression of 14 AMP genes was investigated by qRT-PCR analysis after microbial challenge of the *TmSpz1b*-silenced *T. molitor* larvae. In hemocytes, the expression levels of seven of the 14 AMP genes were positively regulated (i.e., downregulated in *TmSpz1b*-silenced individuals) (Figure 6A). *TmTene1* was decreased by 56% in *E. coli*, 29.4% in *S. aureus*, and 72.7% in *C. albicans* (Figure 6A). The respective decreases for *TmTene3* were 83, 75, and 76% (Figure 6C). The respective decreases for *TmAtt1a* were 82, 83, and 88% (Figure 6E). The respective decreases for *TmAtt1b* were 30, 52, and 50% (Figure 6F). The respective decreases for *TmColeA* were 85, 86, and 88% (Figure 6H). The respective decreases for *TmCole*B were 91, 81, and 83% (Figure 6I). Finally, the respective decreases for *TmDef-like* were 53, 46, and 77% (Figure 6K). In fat bodies, the expression of five AMP genes was positively regulated in *TmSpz1b*-silenced larvae (Figure 7). The respective decreases in *E. coli*, *S. aureus*, and *C. albicans* were 82, 83, and 88% *TmAtt1a* (Figure 7E); 30, 52, and 50% for *TmAtt1b* (Figure 7F); 85, 86, and 88% for *TmColeA* (Figure 7H); 90, 82, and 83% for *TmCole*B (Figure 7I); and 93, 0, and 70% for *TmTLP1* (Figure 7L).

**FIGURE 8:**
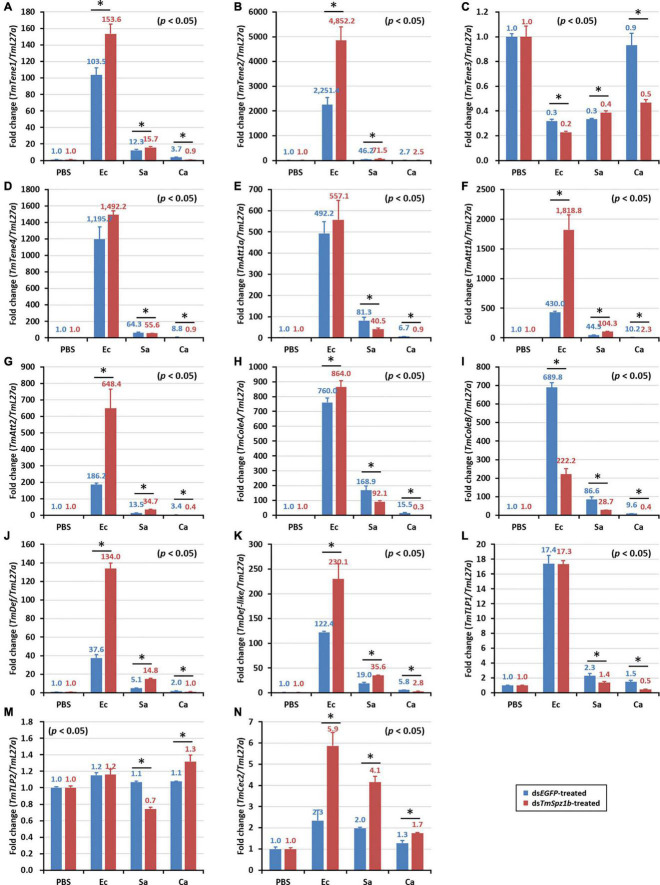
Effects of *TmSpz1b* gene-silencing on the expression of 14 AMP genes in response to pathogen injection in the gut. *E. coli, S. aureus*, and *C. albicans* were injected into ds*TmSpz1b*-treated *T. molitor* larvae. The expression levels of 14 AMP genes were determined at 24-post injection, by qRT-PCR. In the gut, only one AMP gene (*TmColeoptericin2*) was significantly decreased by *TmSpz1b* RNAi. *dsEGFP* was injected as a negative control, and *TmL27a* was used as an internal control. All experiments were performed in triplicate. Asterisks indicate significant differences between ds*TmSpz1b* and ds*EGFP*-treated groups when compared by Student’s *t*-test (*P* < 0.05). *TmTene1* (**A**; *TmTeneecin-1*), *TmTene2* (**B**; *TmTenecin-2*). *TmTene3* (**C**; *TmTenecin-3*), *TmTene4* (**D**; *TmTenecin-4*), *TmAtt1a* (**E**; *TmAttacin-1a*), *TmAtt1b* (**F**; *TmAttacin-1b*), *TmAtt2* (**G**; *TmAttacin-2*), *TmCole1* (**H**; *TmColeoptericin-1*), *TmCole2* (**I**; *TmColeoptericin-2*), *TmDef1* (**J**; *TmDefensin-1*), *TmDef2* (**K**; *TmDefensin-2*), *TmTLP1* (**L**; *TmThaumatin-like protein-1*), *TmTLP2* (**M**; *TmThaumatin-like protein-2*), *TmCec2* (**N**; *TmCecropin-2*).

**FIGURE 9:**
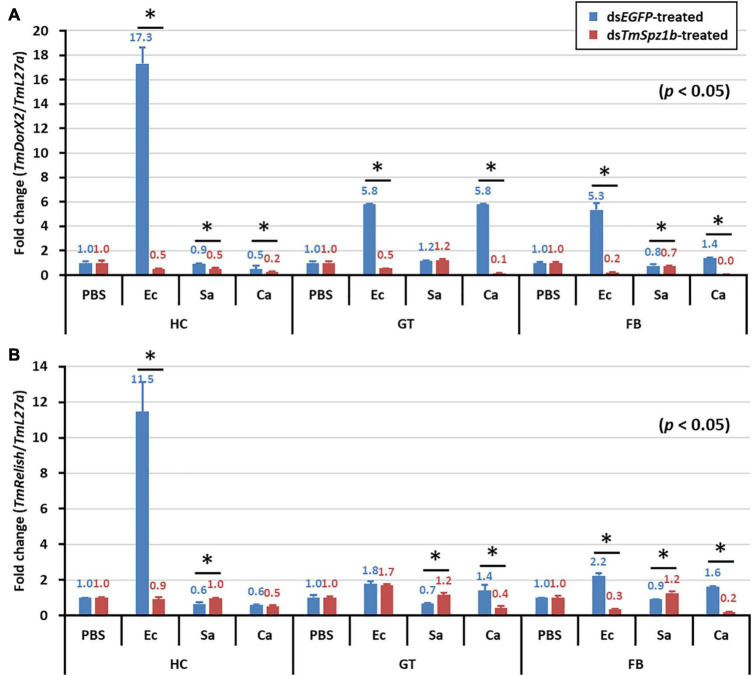
Effect of *TmSpz-1b* silencing on the transcriptional regulation of NF-κB genes after injections of *E. coli, S. aureus*, and *C. albicans.* mRNA expression levels of the NF-κB genes viz. *TmDorX2* **(A)**, and *TmRelish* **(B)** have been investigated by RT-qPCR. Larvae were injected with ds*EGFP* as a negative control, and *TmL27a* expression was assessed as an internal control. All experiments were performed in triplicate. Asterisks indicate significant differences in NF-κB gene expression between the ds*TmSpz-like-* and *dsEGFP-*treated groups when compared by Student’s *t*-test (*P* < 0.05).

**FIGURE 10:**
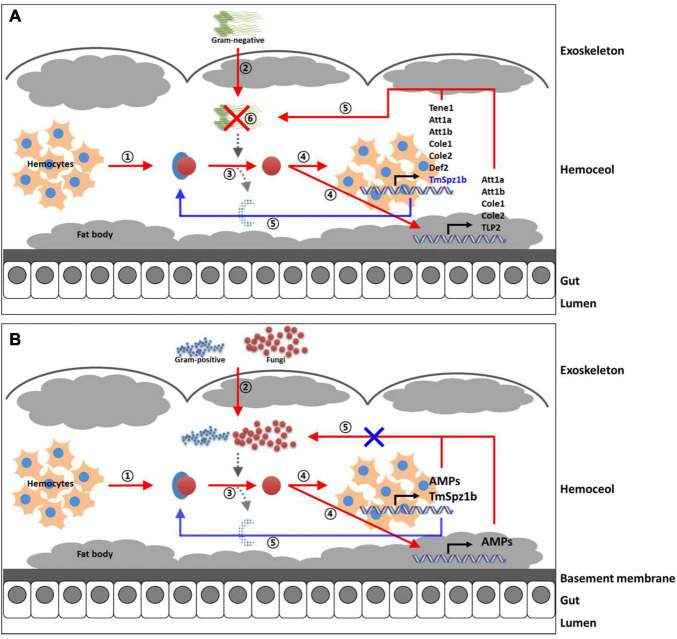
Proposed immunological function of *Tm*Spz1b in innate immune response to microbial challenges. The immunological functions of *Tm*Spz1b against *E. coli* **(A)**, *S. aureus*, and *C. albicans* **(B)** are separately proposed. **(A)** *Tm*Spz1b influences the transcriptional regulation of seven and five AMP genes in hemocytes and fat body of *T. molitor* larvae in killing the Gram-negative pathogen *E. coli.* **(B)** *Tm*Spz1b does not affect the resistance to Gram-positive and fungal infections in *T. molitor* larvae due to non-regulation of AMP genes.

In the gut, significantly decreased expression was detected in only one AMP gene (Figure 8). *TmColeB* was decreased by 68% in *E. coli*, 67% in *S. aureus*, and 96% in *C. albicans* by *TmSpz1b* RNAi (Figure 8I). Further, to delineate the regulatory role of *TmSpz1b* in the Toll/IMD signaling cascade mechanism, we studied the transcriptional regulation of NF-κB factors such as *TmDorX2* (Toll pathway) and *TmRelish* (IMD pathway). The transcriptional levels of *TmDorX2* and *TmRelish* after *TmSpz1b* silencing and challenge of *E. coli, S. aureus*, and *C. albicans* is shown in Figure 9. There was a positive regulation of *TmDorX2* transcripts upon *TmSpz1b* silencing after all microorganisms challenge in hemocytes and fat body tissue while in gut it was observed in case of *E. coli* and *C. albicans* infection (Figure 9A). In *E. coli* challenged individuals, maximum downregulation of *TmDorX2* transcripts were observed under *TmSpz1b* silencing conditions. The *TmRelish* transcripts were mostly found to be negatively regulated in *TmSpz1b* silenced individuals except in case of *E. coli* infection in hemocytes and fat body tissue (Figure 9B). A pertinent observation was that *Tm*Spz1b released from the hemocytes positively regulated *TmTene1, TmAtt1a, TmAtt1b, TmColeA, TmColeB*, and *TmDef-like* in the hemocytes and *TmAtt1a, TmAtt1b, TmColeA, TmColeB*, and *TmTLP2* in the fat bodies. Activity of these genes could possibly kill *E. coli* in hemocoel. Thus, survival of *T. molitor* larvae was improved (Figure 10A). On the contrary, *TmSpz1b* silencing had no impact on the resistance to *S. aureus* and *C. albicans* infections in *T. molitor* larvae (Figure 10B). These observations suggest that *Tm*Spz1b is required to confer antibacterial defense against Gram-negative bacteria and not Gram-positive bacteria and fungi, by regulation of AMPs in hemocytes and fat bodies.

## Discussion

Spätzle protein has an important role in dorsal-ventral polarity in *Drosophila* and invertebrate development. Its immunological role has been characterized in insects as well as aquatic invertebrates (Imler and Hoffmann, 2002). The endogenous *Drosophila* spätzle protein is critical for the activation of the Toll pathway by direct binding to Toll receptors. In the present study, a novel spätzle isoform (*Tm*Spz1b) involved in the *T. molitor* innate immunity was reported, that causes the sequestration of Gram-negative bacteria by the regulatory action of AMPs and enhances the survival of *T. molitor* larvae. Silencing of *TmSpz1b* led to the positive regulation of AMPs in hemocytes and fat bodies of *T. molitor* larvae, suggesting the requirement of *Tm*Spz1b in microbial killing (specifically against *E. coli*) due to action of AMPs in the hemocoel.

Initially, the *TmSpz1b* gene was identified by bioinformatics analysis from *T. molitor* RNA sequencing database. The ORF sequence was confirmed by cloning and sequencing. Domain analysis of *Tm*Spz1b indicated a signal peptide region (indicating secretion to hemocoel), one putative cleavage site, and one cystine-knot domain composed of 93 amino acid residues. A previous study structurally characterized the disulfide-linked cystine-knot dimer by crystallization (Hoffmann et al., 2008b). In addition, the same authors described the cleavage of the pre-form of spätzle protein in *D. melanogaster* by trypsin, and a cystine-knot domain with seven conserved cysteine residues in the C106 active form of spätzle (Hoffmann et al., 2008a). The five homologs of spätzle (spz2 − 6), which had a neurotrophin-like cystine-knot domain, were identified by blast analysis with the *Drosophila* genomic and transcriptomic database (Parker et al., 2001). The cystine-knot domain and the specific cysteine residues involved in the formation of disulfide bridges have also been identified in spätzle proteins of *B. mori* and *M. sexta* (Wang et al., 2007; An et al., 2010). Unlike other insect spätzle proteins, the spätzle protein in Chinese oak silkworm, *A. pernyi* (*Ap*Spz) contains a cystine-knot domain with only two conserved cysteine residues (Sun et al., 2016). The *T. molitor* spätzle protein isoforms such as *Tm*Spz4 and *Tm*Spz6 also contain a cysteine knot domain in their C-terminus with conserved cysteine residues forming disulfide bridges (Edosa et al., 2020a,b). In addition, examination of the spätzle proteins in aquatic invertebrates, such as shrimp and clam, has revealed a conserved cystine-knot domain. The deduced amino acid sequence of Spätzle-like protein identified from the expressed sequence tag of Chinese shrimp, *F. chinensis* (*Fc*Spz), includes a signal peptide region and a cystine-knot domain with seven cysteine residues (Shi et al., 2009). Spätzle proteins from other shrimp, such as *Penaeus monodon* (*Pm*Spz1) and *Litopenaeus vannamei* (*Lv*Spz4), as well as the first mollusk spätzle homolog gene identified from *P. undulate*, also include a cystine-knot domain with seven cysteine residues (Yu et al., 2015; Boonrawd et al., 2017; Yuan et al., 2017). A signal peptide region that is promiscuous in all spätzle proteins enables its transport through cell membranes and secretion to the hemocoel. Consistent with these previous studies, our results indicated that *Tm*Spz1b may be secreted from the cells to the hemocoel and that serine protease (the Spätzle processing enzyme) may activate *Tm*Spz1b. In *Drosophila*, Persephone in response to danger signals and damage associated molecular patterns (DAMPs) are also responsible for cleaving spätzle and seem to be important in differentiating harmful microbes from commensals (Shaukat et al., 2015; Issa et al., 2018). Silencing of Spätzle processing enzyme in *Drosophila* mutants leads to impaired immunity against the Gram-positive bacterium *Enterococcus faecalis* and not the Gram-negative bacterium *Pseudomonas aeruginosa*, suggesting a role of Spätzle processing enzyme in the Toll pathway (Mulinari et al., 2006). Seven cysteine residues may also be involved in the structure formation with a three disulfide bridge and spätzle dimer. Generally, upon cleavage the Spätzle fragments form a dimer held together by intermolecular disulfide bridges (Weber et al., 2003).

To understand the functional role of *Tm*Spz1b in *Tenebrio* innate immune responses against microbial challenge, three different experiments were designed. Although, the expression of *TmSpz1b* mRNA was greater in adults than those in larval stages, we focused our experiments at the larval stage. We hypothesized that knocking down immune genes in the larvae was greater than in adults. Further, the larval stages in *T. molitor* have high industrial value such as food and feed. Under temporal distribution experiments, *TmSpz1b* was found to be induced more in hemocytes 6 h following the injection of *E. coli, S. aureus*, and *C. albicans*. In our previous studies we have used the same pathogens to identify the immunological role of Toll and IMD pathways through RNAi experiments (Keshavarz et al., 2020). In normal conditions, *T. molitor* challenged by these pathogens shows higher survival rate. Upon challenge by the same pathogens in a *TmSpz1b* dsRNA treated larvae, mortality increased. We hypothesized that these pathogens are effective in the functional characterization of *T. molitor* innate immune pathways. Spätzle proteins were initially expressed from the cells and localized in the hemocoel for a rapid response to produce AMPs. This has been studied in lepidopteran insects. In *M. sexta*, the *spätzle* gene was specifically induced by Gram-positive bacterium *M. luteus* in hemocytes (An et al., 2010). Similarly, the *A. pernyi* spätzle (*Ap*Spz) was induced by the Gram-positive bacterium *E. pernyi* and the fungus, *N. pernyi*, but not by the Gram-negative bacterium *E. coli* (Sun et al., 2016). However, in *B. mori*, *BmSpz1* was induced by *E. coli*, *M. luteus*, and fungi *Saccharomyces cerevisiae* (Wang et al., 2007). Furthermore, in the aquatic shrimp *L. vannamei*, *Lv*Spz4 was induced by both *S. aureus* and *V. alginolyticus* (Yuan et al., 2017). In *A. sinica*, the spätzle gene belonging to the spätzle-4 family was induced by *M. lysodeikticus* (Zheng et al., 2012). Interestingly, *PmSpz1* was induced by WSSV (Boonrawd et al., 2017). These results suggest that spätzle genes can be induced by different microorganisms. In the present study, the induction of *TmSpz1b* in response to *E. coli* infection may suggest a signaling cross-talk between the Toll and IMD pathways in *T. molitor*. *T. molitor* spätzle isoforms, such as *TmSpz4* and *TmSpz6*, were temporally induced after *E. coli* infection, suggesting the role of AMPs in killing *E. coli*. Furthermore, based on this background, spätzle genes induced in hemocytes at 6 h post-injection of microorganisms may be involved in the secondary activation of the Toll pathway. It was reported that the spätzle produced from hemocytes, an immune organ, regulates production of AMPs from fat bodies in *Drosophila* (Lavine and Strand, 2002; Shia et al., 2009). Similarly, the hemocytes are the main organs that produce the *Tm*Spz1b protein in the mealworm.

Next, we characterized the effects of *TmSpz1b* RNAi on larval mortality against microbial challenge. *E. coli*, *S. aureus*, or *C. albicans* were injected into ds*TmSpz1b*-treated *T. molitor* larvae. Mortality of larvae injected with *E. coli* was significantly increased in ds*TmSpz1b*-treated *T. molitor*. Thus, *Tm*Spz1b may have a critical immune function against *E. coli* infection. The results of the present study are consistent with our previous findings that *TmSpz6* and *TmSpz4* RNAi also increase larval mortality after *E. coli* challenge (Edosa et al., 2020a,b). Another valid observation is that knockdown of spätzle in the red palm weevil (*Rhynchophorus ferrugineus*) changes the composition of the gut bacteria, suggesting that spätzle might be involved in the homeostasis of the gut microbiota (Muhammad et al., 2020). We have not studied the knockdown of *TmSpz1b* in the gut of *T. molitor* and all our studies are valid for systemic infection in the whole larvae.

Finally, we explored the role of *Tm*Spz1b in the innate immunity of *T. molitor*. The transcriptional regulation of 14 AMP genes were investigated in hemocytes, fat body, and gut tissues of *TmSpz1b*-silenced *T. molitor* larvae after 24 h exposure to *E. coli, S. aureus*, and *C. albicans*. Several of the AMP genes were positively regulated by *Tm*Spz1b in hemocytes and fat bodies in response to *E. coli* challenge, but not *S. aureus* and *C. albicans* challenges. *TmTene1, TmAtt1a, TmAtt1b, TmColeA, TmColeB*, and *TmDef-like* AMPs were positively regulated from hemocytes. *TmAtt1a, TmAtt1b, TmColeA, TmColeB*, and *TmTLP2* AMPs were expressed in the fat bodies of *T. molitor* larvae after *E. coli*, but not *S. aureus* and *C. albicans* challenges in the survival assay. The downregulation of *RfColeoptericin* and *RfDefensin* was also confirmed in *Rhynchophorus ferrugineus* spätzle silenced larvae indicating that their secretion is under the regulation of the *Rf*Spätzle-mediated signaling pathway and was related to the compromising of *R. ferrugineus* innate immunity and maintenance of homeostasis of gut (Muhammad et al., 2020). This is interesting as Defensin secretion in *Drosophila* and Coleoptericin secretion in the cereal weevil *Sitophilus* are IMD-dependent (Tingvall et al., 2001; Maire et al., 2018).

A previous study reported the altered expression of *spätzle1A*, a ligand for the Toll-like receptor, in Rel1-overexpressing or knockout mutants of *A. aegypti*. Furthermore, susceptibility to the entomopathogenic fungus *B. bassiana* was significantly increased in Rel1 knockout mutants (Bian et al., 2005). However, spätzle 5 in *Drosophila* acts as a ligand for the multi-ligand receptor Toll receptor 1, and it is critical in antibacterial immunity against the Gram-positive bacterium *Staphylococcus saprophyticus* and the Gram-negative bacterium *Erwinia carotovora carotovora* 15 (Ecc15) (Nonaka et al., 2018). A co-immunoprecipitation assay indicated that the *M. sexta* Toll receptor (*Ms*Toll) can only bind to activated spätzle protein (C-108), and not inactive spätzle protein. Injection of recombinant C-108 induced several AMP genes, including *drosomycin*, *cecropin*, *attacin*, *moricin*, and *lebocin*, whereas injection of recombinant C-108 after treatment with *Ms*Toll-specific antibody could not activate AMP expression, suggesting that the Toll signaling pathway was activated by binding of the active form of spätzle to the Toll receptor (Zhong et al., 2012). In the present study co-immunoprecipitation or pull-down assays has not been conducted to prove the interaction between TmSpz1b and TmToll receptors. In shrimp, several studies sought to functionally characterize spätzle genes. In one study, *Fc*Spz (*F. chinensis* spätzle) was induced by injection of both *V. anguillarum* and WSSV, and the AMP gene crustin 2 was upregulated by injection of recombinant *Fc*Spz C-114 protein (the active form of *Fc*Spz protein) in crayfish (Shi et al., 2009). In addition, mortality against WSSV was significantly decreased by co-injection of recombinant *Pm*Spz1 protein, and the injection of recombinant *Pm*Spz1 induced the expression of four AMP genes, including *crustinPm1*, *crustinPm7*, *ALFPm3*, and *penaeidin3* (Boonrawd et al., 2017).

The studies on *T. molitor* Spätzle proteins have been fragmentary with individual studies on spätzle isoforms. During previous studies in *T. molitor* model, the extracellular Toll signaling pathway was fully characterized by biochemical studies with purified peptidoglycan from microorganisms (Kim et al., 2008; Roh et al., 2009; Yu et al., 2010). The polymeric diaminopimelic acid (DAP)-type peptidoglycan from Gram-negative bacteria can be recognized by the PGRP-SA complex, which activates spätzle in *T. molitor* (Yu et al., 2010; Keshavarz et al., 2020). In addition, *Tm*Cactin, the downstream component of Toll signaling pathway, plays an important role in innate immune responses against *E. coli* and *S. aureus* by positively regulating seven AMP genes (Jo et al., 2017). *Tm*Toll-7, one of the important Toll receptors, specifically regulates seven AMP genes to clear invading *E. coli* (Park et al., 2019). Prior studies have demonstrated that five AMP genes (*TmTene1*, *TmDef-like*, *TmCole1*, *TmCole2*, and *TmAtt1b*) are mainly regulated by Toll signaling–related genes (*Tm*Spz1b, *Tm*Toll-7, and *Tm*Cactin) against *E. coli* challenge. These five AMP genes are mainly involved in sequestering of *E. coli* in the insect system (Table 2).

**TABLE 2:** Important AMP genes predicted by our recent results.

Gene name	*Tm*Spz1b			*Tm*Cactin	*Tm*Toll-7

Tissues	Hemocytes	Fat body	Gut	Whole body	Whole body
Genes active against *E. coli*	TmTene1 ***Tm*Def-like** ***TmCole*A** ***TmCole*B** *Tm*Att-1a ***Tm*Att-1b**	***TmCole*A** ***TmCole*B** *Tm*Att-1a ***Tm*Att-1b** *TmTLP*1	***TmColeB***	***TmTene*1** *TmTene*4 *Tm*Def ***Tm*Def-like** ***TmCole*A** ***TmCole*B** ***Tm*Att-1b**	***TmTene*1** *Tm*Def ***Tm*Def-like** ***TmCole*A** *Tm*Att-2
References	–	–	–	Jo et al., 2017	Park et al., 2019

*Bold texts mean some AMPs overlapped by different RNAi.*

The collective findings indicate that *Tm*Spz1b activated by *E. coli* positively regulates AMP genes in hemocytes and fat bodies. We propose an immune function of *Tm*Spz1b in the mealworm. Thus, it is possible that *Tm*Spz1b interacts with *Tm*Toll-7. This needs to be studied further using co-immunoprecipitation or pull-down assays. Further, we studied the expression of the NF-kB genes such as *TmDorX2*, and *TmRelish* in *TmSpz-1b* silenced individuals using qRT-PCR to establish the involvement of *TmSpz-1b* in *T. molitor* innate immunity related to the TLR-NF-kB pathway. The positive regulation of *TmDorX2* transcripts in hemocytes, fat body, and gut in *TmSpz1b* silenced individuals upon *E. coli* challenge substantiates the relationship between Spätzle and NF-κB factor Dorsal within the Toll signaling pathway. We also propose that the upregulated AMP genes (negatively regulated) in *TmSpz1b*-silenced model—*TmAtt2* and *TmDef* in hemocytes, *TmTene4*, *TmAtt2*, and *TmDef-like* in fat bodies, and *TmTene1*, *TmTene2*, *TmTene4*, *TmAtt1b*, *TmAtt2*, *TmDef*, *TmDef-like*, and *TmCec2* in the gut—may be induced by another signaling pathway such as the IMD pathway, as well as a Toll signaling pathway induced by another Spätzle protein to maintain homeostasis. Interestingly, however, we have observed negative regulation of *TmTene2* and *TmAtta1a* following silencing of *TmCactin* transcripts and *E. coli* challenge (Jo et al., 2017). In case of *Tene2*, it has been demonstrated that the production of this AMP is triggered by the Toll pathway through recognition of Gram-negative peptidoglycans (Roh et al., 2009; Yu et al., 2010), and an elevation of *Tene2* transcripts after *TmCactin* silencing could be attributed to the IMD signaling pathway. This leads to ask the most pertinent question- whether some AMPs are synergistically turned on by both Toll and IMD pathways. Although the components of IMD pathway in *T. molitor* have been deciphered (Johnston et al., 2013), the IMD pathway is still elusive in this insect model. It would be interesting to note the effect of knockdown of IMD pathway components on IMD pathway and putative AMP gene expression. The information will contribute to the understanding of Toll and IMD pathway regulated AMP gene expression.

## Conclusion

*Tm*Spz1b is involved in the innate immunity of the mealworm beetle, *T. molitor*, by mediating the secretion of several AMPs in the beetle. These AMPs have a direct role in killing the Gram-negative bacterium *E. coli* in the hemocoel and reducing the mortality of *T. molitor* larvae.

## Data Availability Statement

Publicly available datasets were analyzed in this study. This data can be found here: GenBank/XP015840683.1; XP_975083.1.

## Author Contributions

YJ and YH: conceptualization, methodology, visualization, and project administration. YH: software, validation, resources, data curation, supervision, and funding acquisition. YB and YJ: formal analysis. YB, BK, and KP: investigation. YB, TE, MK, and MAK: writing—original draft preparation. BP, YL, and YH: writing—review and editing. All authors have read and agreed to the published version of the manuscript.

## Conflict of Interest

The authors declare that the research was conducted in the absence of any commercial or financial relationships that could be construed as a potential conflict of interest.

## Publisher’s Note

All claims expressed in this article are solely those of the authors and do not necessarily represent those of their affiliated organizations, or those of the publisher, the editors and the reviewers. Any product that may be evaluated in this article, or claim that may be made by its manufacturer, is not guaranteed or endorsed by the publisher.

## Footnotes

1
http://bioinfo.ut.ee/primer3-0.4.0/

2
http://www.cbs.dtu.dk/services/SignalP/

3
www.expasy.org

4
http://primer3plus.com/cgi-bin/dev/primer3plus.cgi

5
http://www.flyrnai.org/cgi-bin/RNAi_find_primers.pl

6
https://blast.ncbi.nlm.nih.gov/Blast.cgi

## References

[B1] Ali Mohammadie Kojour M. Han Y. S. Jo Y. H. (2020). An overview of insect innate immunity. *Entomol. Res.* 50 282–291. 10.1111/1748-5967.12437

[B2] Ali Mohammadie Kojour M. Jang H. A. Edosa T. T. Keshavarz M. Kim B. B. Bae Y. M. (2021). Identification, in silico characterization, and expression analysis of *Tenebrio molitor* Cecropin-2. *Entomol. Res.* 51 74–82. 10.1111/1748-5967.12476

[B3] An C. Jiang H. Kanost M. R. (2010). Proteolytic activation and function of the cytokine Spatzle in the innate immune response of a lepidopteran insect, Manduca sexta. *FEBS J.* 277 148–162. 10.1111/j.1742-4658.2009.07465.x 19968713 PMC2805777

[B4] Anderson K. V. Bokla L. Nusslein-Volhard C. (1985). Establishment of dorsal-ventral polarity in the Drosophila embryo: the induction of polarity by the Toll gene product. *Cell* 42 791–798. 10.1016/0092-8674(85)90275-2 3931919

[B5] Arnot C. J. Gay N. J. Gangloff M. (2010). Molecular mechanism that induces activation of Spatzle, the ligand for the Drosophila Toll receptor. *J. Biol. Chem.* 285 19502–19509. 10.1074/jbc.M109.098186 20378549 PMC2885229

[B6] Bian G. Shin S. W. Cheon H. M. Kokoza V. Raikhel A. S. (2005). Transgenic alteration of Toll immune pathway in the female mosquito Aedes aegypti. *Proc. Natl. Acad. Sci. U S A* 102 13568–13573. 10.1073/pnas.0502815102 16157887 PMC1224621

[B7] Boonrawd S. Mani R. Ponprateep S. Supungul P. Masrinoul P. Tassanakajon A. (2017). Characterization of PmSptzle 1 from the black tiger shrimp Peneaus monodon. *Fish Shellfish Immunol.* 65 88–95. 10.1016/j.fsi.2017.04.005 28400214

[B8] Chae J. H. Kurokawa K. So Y. I. Hwang H. O. Kim M. S. Park J. W. (2012). Purification and characterization of tenecin 4, a new anti-Gram-negative bacterial peptide, from the beetle *Tenebrio molitor*. *Dev. Compar. Immunol.* 36 540–546. 10.1016/j.dci.2011.09.010 22001126

[B9] Chasan R. Anderson K. V. (1989). The role of easter, an apparent serine protease, in organizing the dorsal-ventral pattern of the Drosophila embryo. *Cell* 56 391–400. 10.1016/0092-8674(89)90242-0 2492450

[B10] De Gregorio E. Spellman P. T. Tzou P. Rubin G. M. Lemaitre B. (2002). The Toll and Imd pathways are the major regulators of the immune response in Drosophila. *EMBO J.* 21 2568–2579. 10.1093/emboj/21.11.2568 12032070 PMC126042

[B11] Edosa T. T. Jo Y. H. Keshavarz M. Bae Y. M. Kim D. H. Lee Y. S. (2020a). TmSpz4 plays an important role in regulating the production of antimicrobial peptides in response to *Escherichia coli* and Candida albicans infections. *Int. J. Mol. Sci.* 21:1878. 10.3390/ijms21051878 32182940 PMC7084639

[B12] Edosa T. T. Jo Y. H. Keshavarz M. Bae Y. M. Kim D. H. Lee Y. S. (2020b). TmSpz6 Is Essential for Regulating the Immune Response to *Escherichia coli* and Staphylococcus aureus Infection in *Tenebrio molitor*. *Insects* 11:105. 10.3390/insects11020105 32033290 PMC7074004

[B13] Ferreira A. G. Naylor H. Esteves S. S. Pais I. S. Martins N. E. Teixeira L. (2014). The Toll-Dorsal Pathway Is Required for Resistance to Viral Oral Infection in Drosophila. *Plos Pathogens* 10:e1004507. 10.1371/journal.ppat.1004507 25473839 PMC4256459

[B14] Godfroy J. I. III Roostan M. Moroz Y. S. Korendovych I. V. Yin H. (2012). Isolated Toll-like receptor transmembrane domains are capable of oligomerization. *PLoS One* 7:e48875. 10.1371/journal.pone.0048875 23155421 PMC3498381

[B15] Goel M. K. Khanna P. Kishore J. (2010). Understanding survival analysis: Kaplan-Meier estimate. *Int. J. Ayurveda Res.* 1 274–278. 10.4103/0974-7788.76794 21455458 PMC3059453

[B16] Hoffmann A. Funkner A. Neumann P. Juhnke S. Walther M. Schierhorn A. (2008a). Biophysical characterization of refolded Drosophila Spatzle, a cystine knot protein, reveals distinct properties of three isoforms. *J. Biol. Chem.* 283 32598–32609. 10.1074/jbc.M801815200 18790733

[B17] Hoffmann A. Neumann P. Schierhorn A. Stubbs M. T. (2008b). Crystallization of Spatzle, a cystine-knot protein involved in embryonic development and innate immunity in Drosophila melanogaster. *Acta Crystallogr. Sect. F Struct. Biol. Cryst. Commun.* 64 707–710. 10.1107/S1744309108018812 18678937 PMC2494967

[B18] Hu X. Yagi Y. Tanji T. Zhou S. Ip Y. T. (2004). Multimerization and interaction of toll and spatzle in drosophila. *Proc. Natl. Acad. Sci. U S A* 101 9369–9374. 10.1073/pnas.0307062101 15197269 PMC438983

[B19] Imler J. L. Hoffmann J. A. (2002). Toll receptors in Drosophila: a family of molecules regulating development and immunity. *Curr. Top. Microbiol. Immunol.* 270 63–79. 10.1007/978-3-642-59430-4_4 12467244

[B20] Issa N. Guillaumot N. Lauret E. Matt N. Schaeffer-Reiss C. Van Dorsselaer A. (2018). The circulating protease persephone is an immune sensor for microbial proteolytic activities upstream of the drosophila toll pathway. *Mol. Cell* 69 539–550. 10.1016/j.molcel.2018.01.029 29452635 PMC5823974

[B21] Jang H. A. Park K. B. Kim B. B. Ali Mohammadie Kojour M. Bae Y. M. Baliarsingh S. (2020a). Bacterial but not fungal challenge up-regulates the transcription of *Coleoptericin* genes in *Tenebrio molitor*. *Entomological Research* 50 440–449. 10.1111/1748-5967.12465

[B22] Jang H. A. Park K. B. Kim B. B. Ali Mohammadie Kojour M. Bae Y. M. Baliarsingh S. (2020b). In silico identification and expression analyses of Defensin genes in the mealworm beetle *Tenebrio molitor*. *Entomol. Res.* 50 575–585. 10.1111/1748-5967.12468

[B23] Jang I. H. Chosa N. Kim S. H. Nam H. J. Lemaitre B. Ochiai M. (2006). A Spatzle-processing enzyme required for toll signaling activation in Drosophila innate immunity. *Dev. Cell.* 10 45–55. 10.1016/j.devcel.2005.11.013 16399077

[B24] Jo Y. H. Kim Y. J. Park K. B. Seong J. H. Kim S. G. Park S. (2017). TmCactin plays an important role in Gram-negative and -positive bacterial infection by regulating expression of 7 AMP genes in *Tenebrio molitor*. *Sci. Rep.* 7:46459. 10.1038/srep46459 28418029 PMC5394457

[B25] Jo Y. H. Park S. Park K. B. Noh M. Y. Cho J. H. Ko H. J. (2018). In silico identification, characterization and expression analysis of attacin gene family in response to bacterial and fungal pathogens in *Tenebrio molitor*. *Entomol. Res.* 48 45–54. 10.1111/1748-5967.12287

[B26] Johnston P. R. Makarova O. Rolff J. (2013). Inducible defenses stay up late: temporal patterns of immune gene expression in *Tenebrio molitor*. *G3 (Bethesda)* 4 947–955. 10.1534/g3.113.008516 24318927 PMC4065263

[B27] Jones P. Binns D. Chang H. Y. Fraser M. Li W. McAnulla C. (2014). InterProScan 5: genome-scale protein function classification. *Bioinformatics* 30 1236–1240. 10.1093/bioinformatics/btu031 24451626 PMC3998142

[B28] Kawai T. Akira S. (2010). The role of pattern-recognition receptors in innate immunity: update on Toll-like receptors. *Nat. Immunol.* 11 373–384. 10.1038/ni.1863 20404851

[B29] Keshavarz M. Jo Y. H. Edosa T. T. Bae Y. M. Han Y. S. (2020). TmPGRP-SA regulates antimicrobial response to bacteria and fungi in the fat body and gut of *Tenebrio molitor*. *Int. J. Mol. Sci.* 21:2113. 10.3390/ijms21062113 32204438 PMC7139795

[B30] Kim C. H. Kim S. J. Kan H. Kwon H. M. Roh K. B. Jiang R. (2008). A three-step proteolytic cascade mediates the activation of the peptidoglycan-induced toll pathway in an insect. *J. Biol. Chem.* 283 7599–7607. 10.1074/jbc.M710216200 18195005

[B31] Kim D. H. Lee Y. T. Lee Y. J. Chung J. H. Lee B. L. Choi B. S. (1998). Bacterial expression of tenecin 3, an insect antifungal protein isolated from *Tenebrio molitor*, and its efficient purification. *Mol. Cells* 8 786–789. 9895135

[B32] Kim D. H. Noh M. Y. Park K. B. Jo Y. H. (2017). Expression profiles of two thaumatin-like protein (TmTLP) genes in responses to various micro-organisms from *Tenebrio molitor*. *Entomol. Res.* 47 35–40. 10.1111/1748-5967.12197

[B33] Kumar S. Stecher G. Tamura K. (2016). MEGA7: Molecular Evolutionary Genetics Analysis Version 7.0 for bigger datasets. *Mol. Biol. Evol.* 33 1870–1874. 10.1093/molbev/msw054 27004904 PMC8210823

[B34] Larkin M. A. Blackshields G. Brown N. P. Chenna R. McGettigan P. A. McWilliam H. (2007). Clustal W and Clustal X version 2.0. *Bioinformatics* 23 2947–2948. 10.1093/bioinformatics/btm404 17846036

[B35] Lavine M. Strand M. (2002). Insect hemocytes and their role in immunity. *Insect Biochem. Mol. Biol.* 32 1295–1309. 10.1016/s0965-1748(02)00092-9 12225920

[B36] Lemaitre B. Meister M. Govind S. Georgel P. Steward R. Reichhart J. M. (1995). Functional analysis and regulation of nuclear import of dorsal during the immune response in *Drosophila*. *EMBO J.* 14 536–545. 10.1002/j.1460-2075.1995.tb07029.x 7859742 PMC398111

[B37] Lemaitre B. Nicolas E. Michaut L. Reichhart J. M. Hoffmann J. A. (1996). The dorsoventral regulatory gene cassette spatzle/Toll/cactus controls the potent antifungal response in *Drosophila* adults. *Cell* 86 973–983. 10.1016/s0092-8674(00)80172-5 8808632

[B38] Li H. Li T. Guo Y. Li Y. Zhang Y. Teng N. (2018). Molecular characterization and expression patterns of a non-mammalian toll-like receptor gene (TLR21) in larvae ontogeny of common carp (*Cyprinus carpio* L.) and upon immune stimulation. *BMC Vet. Res.* 14:153. 10.1186/s12917-018-1474-4 29724212 PMC5934810

[B39] Livak K. J. Schmittgen T. D. (2001). Analysis of relative gene expression data using real-time quantitative PCR and the 2(T)(-Delta Delta C) method. *Methods* 25 402–408. 10.1006/meth.2001.1262 11846609

[B40] Maire J. Vincent-Monegat C. Masson F. Zaidman-Remy A. Heddi A. (2018). An IMD-like pathway mediates both endosymbiont control and host immunity in the cereal weevil *Sitophilus spp*. *Microbiome* 6:6. 10.1186/s40168-017-0397-9 29310713 PMC5759881

[B41] Medzhitov R. (2001). Toll-like receptors and innate immunity. *Nat. Rev. Immunol.* 1 135–145. 10.1038/35100529 11905821

[B42] Michel T. Reichhart J. M. Hoffmann J. A. Royet J. (2001). Drosophila Toll is activated by Gram-positive bacteria through a circulating peptidoglycan recognition protein. *Nature* 414 756–759. 10.1038/414756a 11742401

[B43] Morisato D. (2001). Spatzle regulates the shape of the Dorsal gradient in the Drosophila embryo. *Development* 128 2309–2319. 10.1242/dev.128.12.2309 11493550

[B44] Mount D. W. (2007). Using the Basic Local Alignment Search Tool (BLAST). *CSH Protoc. 2007* 2007:db.to17. 10.1101/pdb.top17 21357135

[B45] Muhammad A. Habineza P. Wang X. Xiao R. Ji T. Hou Y. (2020). Spatzle homolog-mediated toll-like pathway regulates innate immune responses to maintain the homeostasis of gut microbiota in the red palm weevil, Rhynchophorus ferrugineus Olivier (Coleoptera: Dryophthoridae). *Front. Microbiol.* 11:846. 10.3389/fmicb.2020.00846 32523559 PMC7261851

[B46] Mulinari S. Hacker U. Castillejo-Lopez C. (2006). Expression and regulation of Spatzle-processing enzyme in Drosophila. *FEBS Lett.* 580 5406–5410. 10.1016/j.febslet.2006.09.009 16996061

[B47] Nakamoto M. Moy R. H. Xu J. Bambina S. Yasunaga A. Shelly S. S. (2012). Virus recognition by Toll-7 activates antiviral autophagy in Drosophila. *Immunity* 36 658–667. 10.1016/j.immuni.2012.03.003 22464169 PMC3334418

[B48] Nie L. Cai S. Y. Shao J. Z. Chen J. (2018). Toll-like receptors, associated biological roles, and signaling networks in non-mammals. *Front. Immunol.* 9:1523. 10.3389/fimmu.2018.01523 30034391 PMC6043800

[B49] Noh M. Y. Jo Y. H. (2016). Identification and sequence analysis of two thaumatin-like protein (TmTLP) genes from *Tenebrio molitor*. *Entomol. Res.* 46 354–359. 10.1111/1748-5967.12198

[B50] Nonaka S. Kawamura K. Hori A. Salim E. Fukushima K. Nakanishi Y. (2018). Characterization of Spz5 as a novel ligand for Drosophila Toll-1 receptor. *Biochem. Biophys. Res. Commun.* 506 510–515. 10.1016/j.bbrc.2018.10.096 30361090

[B51] Park S. Jo Y. H. Park K. B. Ko H. J. Kim C. E. Bae Y. M. (2019). TmToll-7 plays a crucial role in innate immune responses against gram-negative bacteria by regulating 5 AMP genes in *Tenebrio molitor*. *Front. Immunol.* 10:310. 10.3389/fimmu.2019.00310 30930888 PMC6424196

[B52] Parker J. S. Mizuguchi K. Gay N. J. (2001). A family of proteins related to Spatzle, the toll receptor ligand, are encoded in the Drosophila genome. *Proteins* 45 71–80. 10.1002/prot.1125 11536362

[B53] Roh K. B. Kim C. H. Lee H. Kwon H. M. Park J. W. Ryu J. H. (2009). Proteolytic cascade for the activation of the insect toll pathway induced by the fungal cell wall component. *J. Biol. Chem.* 284 19474–19481. 10.1074/jbc.M109.007419 19473968 PMC2740573

[B54] Schneider D. S. Jin Y. Morisato D. Anderson K. V. (1994). A processed form of the Spatzle protein defines dorsal-ventral polarity in the Drosophila embryo. *Development* 120 1243–1250. 10.1242/dev.120.5.1243 8026333

[B55] Shaukat Z. Liu D. Gregory S. (2015). Sterile inflammation in *Drosophila*. *Mediat. Inflamm.* 2015:369286. 10.1155/2015/369286 25948885 PMC4408615

[B56] Shi X. Z. Zhang R. R. Jia Y. P. Zhao X. F. Yu X. Q. Wang J. X. (2009). Identification and molecular characterization of a Spatzle-like protein from Chinese shrimp (*Fenneropenaeus chinensis*). *Fish Shellfish Immunol.* 27 610–617. 10.1016/j.fsi.2009.07.005 19616633

[B57] Shia A. K. Glittenberg M. Thompson G. Weber A. N. Reichhart J. M. Ligoxygakis P. (2009). Toll-dependent antimicrobial responses in Drosophila larval fat body require Spatzle secreted by haemocytes. *J. Cell. Sci.* 122 4505–4515. 10.1242/jcs.049155 19934223 PMC2787462

[B58] Shin S. W. Bian G. Raikhel A. S. (2006). A toll receptor and a cytokine, Toll5A and Spz1C, are involved in toll antifungal immune signaling in the mosquito Aedes aegypti. *J. Biol. Chem.* 281 39388–39395. 10.1074/jbc.M608912200 17068331

[B59] Sun Y. Jiang Y. Wang Y. Li X. Yang R. Yu Z. (2016). The toll signaling pathway in the Chinese oak silkworm, antheraea pernyi: innate immune responses to different microorganisms. *PLoS One* 11:e0160200. 10.1371/journal.pone.0160200 27483463 PMC4970820

[B60] Tingvall T. O. Roos E. Engstrom Y. (2001). The imd gene is required for local Cecropin expression in Drosophila barrier epithelia. *Embo Rep.* 2 239–243. 10.1093/embo-reports/kve048 11266367 PMC1083844

[B61] Vaniksampanna A. Longyant S. Charoensapsri W. Sithigorngul P. Chaivisuthangkura P. (2019). Molecular isolation and characterization of a spatzle gene from *Macrobrachium rosenbergii*. *Fish Shellfish Immunol.* 84 441–450. 10.1016/j.fsi.2018.10.015 30308293

[B62] Wang Y. Cheng T. C. Rayaprolu S. Zou Z. Xia Q. Y. Xiang Z. H. (2007). Proteolytic activation of pro-spatzle is required for the induced transcription of antimicrobial peptide genes in lepidopteran insects. *Dev. Compar. Immunol.* 31 1002–1012. 10.1016/j.dci.2007.01.001 17337053 PMC2020823

[B63] Weber A. N. R. Tauszig-Delamasure S. Hoffmann J. A. Lelievre E. Gascan H. Ray K. P. (2003). Binding of the Drosophila cytokine Spatzle to Toll is direct and establishes signaling. *Nat. Immunol.* 4 794–800. 10.1038/ni955 12872120

[B64] Yamamoto-Hino M. Goto S. (2016). Spatzle-Processing Enzyme-independent Activation of the Toll Pathway in Drosophila Innate Immunity. *Cell. Struct. Funct.* 41 55–60. 10.1247/csf.16002 26843333

[B65] Yang Y. T. Lee M. R. Lee S. J. Kim S. Nai Y. S. Kim J. S. (2017). *Tenebrio molitor* Gram-negative-binding protein 3 (TmGNBP3) is essential for inducing downstream antifungal Tenecin 1 gene expression against infection with Beauveria bassiana JEF-007. *Insect Sci*. 25 969–977. 10.1111/1744-7917.12482 28544681

[B66] Yu D. Wu Y. Xu L. Fan Y. Peng L. Xu M. (2016). Identification and characterization of toll-like receptors (TLRs) in the Chinese tree shrew (Tupaia belangeri chinensis). *Dev. Comp. Immunol.* 60 127–138. 10.1016/j.dci.2016.02.025 26923770

[B67] Yu M. Zhang Y. Tang X. Ren J. Zhang Y. (2015). The first mollusk spatzle homolog gene in the clam, Paphia undulate. *Fish Shellfish Immunol.* 47 712–716. 10.1016/j.fsi.2015.10.017 26477575

[B68] Yu Y. Park J. W. Kwon H. M. Hwang H. O. Jang I. H. Masuda A. (2010). Diversity of innate immune recognition mechanism for bacterial polymeric meso-diaminopimelic acid-type peptidoglycan in insects. *J. Biol. Chem.* 285 32937–32945. 10.1074/jbc.M110.144014 20702416 PMC2963372

[B69] Yuan K. Yuan F. H. Weng S. P. He J. G. Chen Y. H. (2017). Identification and functional characterization of a novel Spatzle gene in Litopenaeus vannamei. *Dev. Comp. Immunol.* 68 46–57. 10.1016/j.dci.2016.11.016 27884706

[B70] Zheng L. P. Hou L. Yu M. Li X. Zou X. Y. (2012). Cloning and the expression pattern of Spatzle gene during embryonic development and bacterial challenge in Artemia sinica. *Mol. Biol. Rep.* 39 6035–6042. 10.1007/s11033-011-1417-7 22203485

[B71] Zhong X. Xu X. X. Yi H. Y. Lin C. Yu X. Q. (2012). A Toll-Spatzle pathway in the tobacco hornworm, Manduca sexta. *Insect Biochem. Mol. Biol.* 42 514–524. 10.1016/j.ibmb.2012.03.009 22516181 PMC3361650

[B72] Zhu J. Y. Wu G. X. Zhang Z. (2014). Upregulation of coleoptericin transcription in *Tenebrio molitor* parasitized by Scleroderma guani. *J. Asia-Pacific Entomol.* 17 339–342. 10.1016/j.aspen.2014.03.001

